# In vitro and in vivo characterization of three *Cellvibrio japonicus* glycoside hydrolase family 5 members reveals potent xyloglucan backbone-cleaving functions

**DOI:** 10.1186/s13068-018-1039-6

**Published:** 2018-02-17

**Authors:** Mohamed A. Attia, Cassandra E. Nelson, Wendy A. Offen, Namrata Jain, Gideon J. Davies, Jeffrey G. Gardner, Harry Brumer

**Affiliations:** 1 Michael Smith Laboratories, University of British Columbia, 2185 East Mall, Vancouver, BC V6T 1Z4 Canada; 2 Department of Chemistry, University of British Columbia, 2036 Main Mall, Vancouver, BC V6T 1Z1 Canada; 3 Department of Biological Sciences, University of Maryland, Baltimore County, Baltimore, MD 21250 USA; 4 Department of Chemistry, University of York, Heslington, York, YO10 5DD UK; 5 Department of Biochemistry and Molecular Biology, University of British Columbia, 2350 Health Sciences Mall, Vancouver, BC V6T 1Z3 Canada; 6 Department of Botany, University of British Columbia, 6270 University Blvd., Vancouver, BC V6T 1Z4 Canada

**Keywords:** Xyloglucan, Saccharification, Glycoside hydrolase, *Cellvibrio japonicus*, Saprophyte

## Abstract

**Background:**

Xyloglucan (XyG) is a ubiquitous and fundamental polysaccharide of plant cell walls. Due to its structural complexity, XyG requires a combination of backbone-cleaving and sidechain-debranching enzymes for complete deconstruction into its component monosaccharides. The soil saprophyte *Cellvibrio japonicus* has emerged as a genetically tractable model system to study biomass saccharification, in part due to its innate capacity to utilize a wide range of plant polysaccharides for growth. Whereas the downstream debranching enzymes of the xyloglucan utilization system of *C. japonicus* have been functionally characterized, the requisite backbone-cleaving *endo*-xyloglucanases were unresolved.

**Results:**

Combined bioinformatic and transcriptomic analyses implicated three glycoside hydrolase family 5 subfamily 4 (GH5_4) members, with distinct modular organization, as potential keystone *endo*-xyloglucanases in *C. japonicus*. Detailed biochemical and enzymatic characterization of the GH5_4 modules of all three recombinant proteins confirmed particularly high specificities for the XyG polysaccharide versus a panel of other cell wall glycans, including mixed-linkage beta-glucan and cellulose. Moreover, product analysis demonstrated that all three enzymes generated XyG oligosaccharides required for subsequent saccharification by known *exo*-glycosidases. Crystallographic analysis of GH5D, which was the only GH5_4 member specifically and highly upregulated during growth on XyG, in free, product-complex, and active-site affinity-labelled forms revealed the molecular basis for the exquisite XyG specificity among these GH5_4 enzymes. Strikingly, exhaustive reverse-genetic analysis of all three GH5_4 members and a previously biochemically characterized GH74 member failed to reveal a growth defect, thereby indicating functional compensation in vivo, both among members of this cohort and by other, yet unidentified, xyloglucanases in *C. japonicus*. Our systems-based analysis indicates distinct substrate-sensing (GH74, GH5E, GH5F) and attack-mounting (GH5D) functions for the *endo*-xyloglucanases characterized here.

**Conclusions:**

Through a multi-faceted, molecular systems-based approach, this study provides a new insight into the saccharification pathway of xyloglucan utilization system of *C. japonicus*. The detailed structural–functional characterization of three distinct GH5_4 *endo*-xyloglucanases will inform future bioinformatic predictions across species, and provides new CAZymes with defined specificity that may be harnessed in industrial and other biotechnological applications.

**Electronic supplementary material:**

The online version of this article (10.1186/s13068-018-1039-6) contains supplementary material, which is available to authorized users.

## Background

Renewable plant biomass is envisioned as a promising alternative to fossil petroleum for the production of liquid fuels and high-value chemicals [1, 2]. Plant cell walls are, however, chemically and structurally complex in nature and require harsh thermo-chemical treatment to yield fermentable sugars. Such processes often generate undesirable by-products that inhibit subsequent microbial conversion [3]. In light of their ability to catalyze the degradation of recalcitrant plant cell walls under ambient conditions, enzymes from saprophytic micro-organisms constitute an attractive palette of biocatalysts for improved biomass saccharification [4]. The discovery and characterization of new enzymes from saprophytes is thus central to advancing biotechnology and, not least, underpins fundamental understanding of the biological roles of these micro-organisms in the global carbon cycle.

The Gram-negative bacterium, *Cellvibrio japonicus* Ueda107 (formerly, *Pseudomonas fluorescens* subsp. *cellulosa)* has emerged as a model saprophytic micro-organism with a demonstrated ability to utilize nearly all plant cell wall polysaccharides, including cellulose, xylans, mannans, arabinans, and pectins [5, 6]. Indeed, sequencing of the *C. japonicus* genome in 2008 revealed vast array of carbohydrate-active enzymes (CAZymes [7]) predicted to be involved in plant cell wall saccharification [8]. The recent development of genome editing techniques for *C. japonicus* has further advanced the biology and bioengineering of this bacterium in biomass conversion [9–13].

**Fig. 1:**
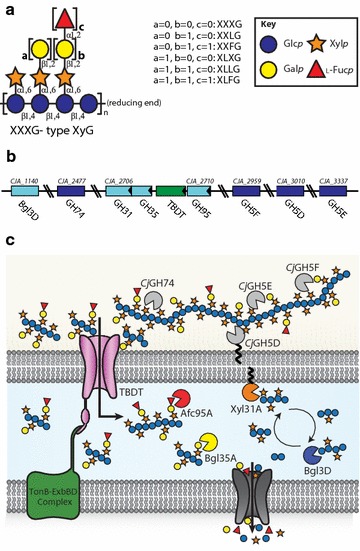
Xyloglucan (XyG) and the xyloglucan utilization system in *C. japonicus*. **a** Structure of dicot XXXG-type fucogalacto-XyG. XyG substructure nomenclature is according to [20]. **b** *C. japonicus* genes involved in XyG utilization. Genes encoding backbone-cleaving *endo*-xyloglucanases (GH5 and GH74) are indicated in navy blue, genes encoding side-chain-cleaving *exo*-glycosidases (GH35 β-galactosidases; GH31 α-xylosidases and GH95 α-l-fucosidase) are in cyan, and the TonB dependent transporter (TBDT) is shown in green. **c** Spatial model of XyG utilization in *C. japonicus*

The xyloglucans (XyG) comprise an important family of cell wall matrix polysaccharides, which are ubiquitous and abundant across the plant kingdom [14, 15]. In dicots, XyGs may constitute up to 25% of the primary cell wall dry-weight, with lower amounts found in conifers (10%) and grasses (< 5%) [16, 17]. Structurally, XyGs have brush-like architectures built upon a linear, cellulosic β(1→4)-d-glucan backbone that is extensively branched with α(1→6)-xylopyranosyl residues at regular intervals. Further elaboration of these branch points with diverse monosaccharides and acetyl groups is dependent on the species and tissue of origin [18, 19]; presently ca. 20 distinct sidechain saccharide compositions are known [20, 21]. The structure of the canonical dicot (fucogalacto)xyloglucan is shown in Fig. 1a. Due to this structural complexity, complete XyG saccharification requires the concerted action of numerous backbone-cleaving *endo*-xyloglucanases and side-chain-cleaving *exo*-glycosidases [22, 23].

As part of our ongoing effort to elucidate the xyloglucan (XyG) utilization system of *C. japonicus*, we functionally characterized a multi-gene XyG utilization locus (XyGUL) in the *C. japonicus* genome via a combination of genetics, enzymology, and structural biology. This XyGUL encodes the three *exo*-glycosidases required for (fucogalacto)xyloglucan sidechain cleavage (a GH95 α-l-fucosidase, a GH35 β-galactosidase, and a GH31 α-xylosidase) together with a predicted TonB-dependent transporter (TBDT) (Fig. 1b, c) [13, 24, 25]. A highly specific β-glucosidase, Bgl3D, which is encoded elsewhere in the genome, works in concert with the *exo*-glycosidases of the XyGUL to effect the complete saccharification of XyG oligosaccharides (XyGOs) in the periplasm (Fig. 1b, c) [26]. Noting that this locus likewise lacked an associated *endo*-xyloglucanase, we also provided biochemical and structural evidence that the lone, secreted *C. japonicus* GH74 member (Fig. 1b, c) could efficiently generate the Glc_4_-based XyGOs required by the downstream *exo*-glycosidases [27].

As we now show, genetic deletion of this GH74 *endo*-xyloglucanase did not, however, impede the growth of *C. japonicus* on the polysaccharide, which suggested the involvement of additional, unidentified *endo*-xyloglucanases. Hence, we also explored the in vitro and in vivo function of three candidate *endo*-xyloglucanases from GH5 subfamily 4 (GH5_4) [28], guided by bioinformatic and transcriptomic analyses. Utilizing a combination of reverse genetics, enzymology, and structural biology, the present study provides a new insight into the upstream deconstruction of XyG.

## Results and discussion

### Transcriptomic analysis reveals a potential keystone *endo*-xyloglucanase from Glycoside Hydrolase (GH) family 5, subfamily 4

We previously showed via quantitative PCR (qPCR) that the *C. japonicus* gene cluster containing *xyl31A* (CJA_2706), *bgl35A* (CJA_2707), CJA_2709, and *afc95A* (CJA_2710) (Fig. 1b), was up-regulated during growth on xyloglucan-containing medium [13]. Biochemical characterization confirmed that *xyl31A*, *bgl35A*, and *afc95A* encode a XyGO-specific GH31 α-xylosidase, GH35 β-galactosidase, and GH95 α-l-fucosidase, respectively, while CJA_2709 was predicted to encode a TonB-dependent transporter (TBDT) [13, 24, 25]. To aid identification of potential *C. japonicus endo*-xyloglucanases acting upstream of these enzymes, a comprehensive expression analysis via RNAseq was performed in the present study. Samples were collected from both exponentially growing and stationary phase cells grown on glucose or xyloglucan as the sole carbon source to allow for analyses of gene expression based on early-stage substrate detection (Additional file 1: Figure S1), late-stage substrate detection (Additional file 1: Figure S2A), or growth rate (Additional file 1: Figure S2B).

During exponential growth, there were 27 CAZyme-encoding genes significantly up-regulated on XyG, including the four genes of the *C. japonicus* XyG cluster, which corroborated previous qPCR results (Additional file 1: Table S1). Notably, CJA_3010, which encodes a GH5 subfamily 4 (GH5_4) member previously annotated as *cel5D*, was the highest upregulated gene followed by the XyG cluster genes CJA_2709 (encoding a predicted TBDT) and CJA_2706 (*xyl31A*, encoding a GH31 α-xylosidase) [8]. Among the large and functionally diverse GH5 family [28], subfamily 4 is the only subfamily known to contain predominant *endo*-xyloglucanases [23], which suggested a keystone role for this enzyme in xyloglucan utilization by *C. japonicus*. Notably, CJA_2477 (previously annotated as *gly74* [8]; Fig. 1b) was not significantly up-regulated during growth on XyG, despite the encoded GH74 *endo*-xyloglucanase being previously shown to have high, specific activity for this polysaccharide [27]. Instead, CJA_2477 appeared to be constitutively expressed at a low level (RPKM levels in the 100–200 range), as were 14 other predicted CAZyme-encoding genes (Additional file 1: Table S2).

The remaining CAZyme genes up-regulated during exponential growth are predicted to have roles in the degradation of a diverse set of polysaccharides, which suggests that there is complex cross-regulation of expression. As xyloglucan is unlikely to be encountered alone during the saprophytic growth habit of *C. japonicus*, these results are suggestive of xyloglucan degradation being one component of a sophisticated plant cell wall degradation response. Congruently, when comparing the stationary phase *C. japonicus* cells growing on XyG and glucose, only two genes of the XyG cluster, *bgl35A* and *afc95A* were still up-regulated on XyG, together with 33 additional predicted and confirmed hemicellulase- and pectinase-encoding genes (Additional file 1: Figure S2A, Table S3). Additionally, when comparing the exponential phase to the stationary phase for xyloglucan-grown cells, we observed that there was a growth-phase-dependent response manifested as a significant shift in the suite of expressed CAZyme genes (Additional file 1: Figure S2B, Table S4). Specifically, these differentially expressed CAZyme genes were not predicted to be XyG-specific, based on their CAZy family membership, which suggested they are part of regulatory circuit that responds generally to polysaccharides. Similar growth-phase-dependent responses have been previously observed during cellulose utilization by *C. japonicus* [12] and *Clostridium thermocellum* (now *Ruminiclostridium thermocellum*) [29, 30].

### Bioinformatic analysis and recombinant production of GH5_4 members from *C. japonicus*

Spurred on by the implication of the GH5 subfamily 4 (GH5_4) member encoded by CJA_3010 in xyloglucan utilization by *C. japonicus*, we searched the genome for potential homologs. *C. japonicus* encodes 15 GH5 members, of which only three belong to subfamily 4 ([8] see http://www.cazy.org/b776.html): the aforementioned CJA_3010 (GenBank ACE84905.1, previously annotated as *cel5D* [8]), CJA_3337 (GenBank ACE83841.1, previously annotated as *cel5E* [8]), and CJA_2959 (GenBank ACE86198.1, previously annotated as *cel5F* [8]). Protein sequence analysis revealed that each of these gene products had a unique, multi-modular architecture that suggested the possibility of distinct cellular localization and biological function (Additional file 1: Figure S3). Considering the lack of demonstrable activity on cellulose and high activity on xyloglucan (vide infra), the corresponding encoded enzymes are referred to as *Cj*GH5D, *Cj*GH5E, and *Cj*GH5F hereafter.

The highly up-regulated CJA_3010 encodes a signal peptidase II lipoprotein signal peptide (predicted by LipoP 1.0 [31]), followed by a serine-rich linker and a GH5_4 catalytic module, and was thus predicted to be anchored extracellularly in the outer membrane by N-terminal cysteine lipidation (Additional file 1: Figure S3). CJA_3337 encodes an N-terminal signal peptide (predicted by SignalP 4.0 [32]) and two carbohydrate-binding modules (CBMs [33]), CBM2 and CBM10, in train with a GH5_4 catalytic module (Additional file 1: Figure S3). CJA_2959 encodes a signal peptide (predicted by SignalP 4.0 [32] a Fibronectin type III (FN3) domain, an undefined region, and a C-terminal GH5_4 catalytic module (Additional file 1: Figure S3). The presence of signal peptides, and CBMs in the case of *Cj*GH5E, is indicative of extracellular secretion of both *Cj*GH5E and *Cj*GH5F.

Amino acid alignment of the catalytic modules of *Cj*GH5D, *Cj*GH5E, and *Cj*GH5F demonstrate conservation of the two catalytic glutamate residues, but low to moderate overall sequence conservation (26–45% identity). Notably, alignment with *endo*-xyloglucanases from *Bacteroides ovatus* [22], *Paenibacillus pabuli* [34], and a rumen metagenome [35] suggests that the *C. japonicus* proteins are members of subfamily 4 (Additional file 1: Figure S4, Table S5). GH5_4 is one of the largest GH5 subfamilies and contains, in addition to specific *endo*-xyloglucanases, promiscuous *endo*-β(1,4)-glucanases, strict cellulases, and mixed-linkage *endo*-β(1,3)/β(1,4)glucanases (reviewed in [23, 28]). As such, we undertook the recombinant production and enzymological characterization of the three *C. japonicus* GH5_4 members to precisely define their catalytic activities in the context of potential biological function.

Our initial attempts to produce the full-length, multi-modular proteins recombinantly in *E. coli* by replacement of the native signal peptides with an N-terminal hexahistidine (His_6_) purification tag were consistently unsuccessful: intact protein mass spectrometry revealed proteolytic instability of His_6_-SRL-GH5D, while His_6_-CBM2-CBM10-GH5E and His_6_-FN3-GH5F had very poor production yields (data not shown). In contrast, His_6_-GH5D (Additional file 1: Figure S3A) was produced as a stable, intact, active protein (calculated mass, 44,222.2 Da; observed by ESI–MS, 44,222.6 Da) in excellent yield (150 mg L^−1^). Likewise, our attempts to produce the individual catalytic modules of *Cj*GH5E and *Cj*GH5F as N-terminally His_6_-tagged constructs (Additional file 1: Figure S3B, C) were successful (His_6_-*Cj*GH5E calculated mass, 41,367.1 Da; observed by ESI–MS, 41,370.1 Da, His_6_-*Cj*GH5F calculated mass, 40,253.8 Da; observed by ESI–MS 40,253.9 Da) with approximate production yields of 14 and 9 mg L^−1^, respectively.

### *Cj*GH5_4 enzymes are highly efficient, specific *endo*-xyloglucanases

Informed by the subfamily membership of the three GH5_4 members, we anticipated that these enzymes might exhibit significant *endo*-hydrolytic activity towards XyG. Hence, this polysaccharide was used to determine pH and temperature optima. *Cj*GH5D, *Cj*GH5E, and *Cj*GH5F each exhibited approximately bell-shaped pH profiles, with the highest activity achieved in 50 mM phosphate buffer (pH 7.5 in the case of *Cj*GH5D and *Cj*GH5E, and pH 7.0 in the case of *Cj*GH5F; Additional file 1: Figure S5). When the three enzymes were incubated with XyG at different temperatures over the course of 10 min, the optimum temperatures were identified as 50 °C (*Cj*GH5D and *Cj*GH5F) and 55 °C (*Cj*GH5E) (Additional file 1: Figure S5). To determine substrate specificity of the three GH5_4 members, a panel of nine soluble polysaccharide substrates were screened under these optimal conditions. Indeed *Cj*GH5D, *Cj*GH5E, and *Cj*GH5F all displayed high specific activity toward XyG (Table 1). No detectable activity toward barley mixed-linkage 1,3/1,4-β-glucan, guar galactomannan, konjac glucomannan, beechwood xylan, wheat flour arabinoxylan, or xanthan for any of the three enzymes was observed. *Cj*GH5D appeared to strictly require the branched XyG structure, while *Cj*GH5E demonstrated trace activities against the artificial 1,4-β-glucans hydroxyethylcellulose (HEC) and carboxymethylcellulose (CMC) at the highest tested substrate concentration (2 mg mL^−1^); specific activities were 200 and 1500-fold less than XyG, respectively (Table 1). Similarly, *Cj*GH5F was able to hydrolyze HEC with an 800-fold lower specific activity than XyG, while no activity towards CMC was detected. Michaelis–Menten analysis for XyG further underscored the high XyG specificity of the three enzymes: remarkably low *K*_m_ values were observed and high *k*_cat_ values recapitulated those previously observed for predominant *endo*-xyloglucanases, including *Cj*GH74 [22, 27, 36, 37] (Table 1, Additional file 1: Figure S6).

**Table 1:** Activity of *Cj*GH5_4 enzymes against different polysaccharide substrates

Enzyme catalytic domains	Substrate	*K*_*m*_ mg mL^−1^	*k*_cat_ s^−1^	Specific activity µmol (min mg)^−1^
*Cj*GH5D	XyG	< 0.025	30.3 ± 0.4	43.3 ± 1.9
*Cj*GH5E	XyG	0.020 ± 0.002	10.3 ± 0.1	15.1 ± 0.1
	Hydroxyethylcellulose (HEC)	ND	ND	0.070 ± 0.004
	Carboxymethylcellulose (CMC)	ND	ND	0.010 ± 0.002
*Cj*GH5F	XyG	0.040 ± 0.003	52.4 ± 0.8	74.8 ± 4.1
	Hydroxyethylcellulose (HEC)	ND	ND	0.090 ± 0.003

Assays conducted at pH 7.5 (*Cj*GH5D and *Cj*GH5E) or pH 7 (*Cj*GH5F). Recombinant enzymes were incubated at 50 °C (*Cj*GH5D and *Cj*GH5F) or 55 °C (*Cj*GH5E) with the different tested substrates

*ND* not determined due to poor specific activity

Time-course analyses of native XyG polysaccharide hydrolysis products by HPAEC-PAD revealed that all three GH5_4 enzymes generated products of intermediate retention time in the early stages of the reactions, with no significant generation of the Glc_4_-based XXXG, XLXG, XXLG, and XLLG limit-digest products (Additional file 1: Figures S7, S8; cf. Fig. 1). These results indicate that the three enzymes hydrolyze XyG through a dissociative, rather than processive [38] mechanism, and are thus canonical *endo*-xyloglucanases (EC 3.2.1.151; cf. EC 3.2.1.150, EC 3.2.1.155). The limit-digest products further revealed that all *C. japonicus* GH5_4 enzymes specifically catalyze hydrolysis at the anomeric position of the unbranched glucose residues of the (galacto)XyG polysaccharide chain (Fig. 1). This cleavage pattern is typical for many GH5 [22, 34, 37], GH9 [39, 40], GH12 [34, 41–44], GH16 [45] and GH74 [46–50] *endo*-xyloglucanases, although certain GH5 [35, 36], GH7 [51], GH44 [40, 52] and GH74 [53–55] members preferentially hydrolyze the XyG backbone between branched glucosyl residues. The canonical XXXG-type XyGOs produced by *Cj*GH5D, *Cj*GH5E, and *Cj*GH5F are direct substrates for the *exo*-glycosidases of the XyG gene cluster [13].

With knowledge of the cleavage specificity of the GH5_4 members, we determined kinetic parameters for the hydrolysis of a panel of chromogenic oligosaccharides to reveal the contribution of side chain substitution on substrate recognition and catalysis (Table 2). All three enzymes were only weakly active on 2-chloro-4-nitrophenyl cellotrioside (GGG-β-CNP) and 2-chloro-4-nitrophenyl cellotetraoside (GGGG-β-CNP), with meagre increases in *k*_cat_/*K*_m_ values arising from the addition of potential − 4 subsite binding for the cellotetraoside (Table 2, Additional file 1: Figure S9) (GH subsite nomenclature according to [56]). Strikingly, the addition of three α(1→6)-xylopyranosyl residues to the glucan backbone resulted in significant increases in catalytic efficiency for all GH5_4 members, which was manifested as 65-, 700-, and 150-fold higher *k*_cat_/*K*_m_ values for XXXG-β-CNP vis-à-vis GGGG-β-CNP with *Cj*GH5D, *Cj*GH5E, and *Cj*GH5F, respectively (Table 2, Additional file 1: Figure S9). These values correspond to 11, 18, and 13 kJ/mol, respectively, of additional transition state stabilization in the formation of the covalent glycosyl-enzyme in these anomeric-configuration-retaining GH5 enzymes (calculated using the formula: ΔΔ*G*^*‡*^ = − *RT* ln[(*k*_cat_/*K*_m_ XXXG)/(*k*_cat_/*K*_m_ GGGG)]) [57]. With XLLG-β-CNP, the specificity constants (*k*_cat_/*K*_m_) were only increased by 1.5 to five fold for the three *endo*-xyloglucanases, thereby indicating that extending β(1→2)-galactopyranosyl residues (Fig. 1) have little additional effect on catalysis (Table 2).

**Table 2:** Kinetic parameters of *Cj*GH5_4 enzymes for (xylo)gluco-oligosaccharide glycosides

Enzyme catalytic domains	Substrate	*K*_m_ mM	*k*_cat_ min^−1^	*k*_cat_*/K*_m_ min^−1^ mM^−1^
*Cj*GH5D	GGG-CNP	ND	ND	2.21 ± 0.05
	GGGG-CNP	ND	ND	5.36 ± 0.07
	XXXG-CNP	0.81 ± 0.10	281 ± 12	347 ± 45
	XLLG-CNP	0.18 ± 0.02	162 ± 4	900 ± 103
*Cj*GH5E	GGG-CNP	11.8 ± 0.6	191 ± 7	16.2 ± 1.0
	GGGG-CNP	5.02 ± 0.35	180 ± 7	35.9 ± 2.8
	XXXG-CNP	0.010 ± 0.001	254 ± 4	(25.4 ± 2.6) × 10^3^
	XLLG-CNP	0.010 ± 0.001	332 ± 9	(33.2 ± 3.4) × 10^3^
*Cj*GH5F	GGG-CNP	ND	ND	6.45 ± 0.30
	GGGG-CNP	ND	ND	16.5 ± 1.0
	XXXG-CNP	0.07 ± 0.01	169 ± 4	(2.41 ± 0.35) × 10^3^
	XLLG-CNP	0.030 ± 0.002	393 ± 8	(13.1 ± 0.9) × 10^3^

*ND* not determined due to limited availability of substrate

### Covalent labelling of *Cj*GH5D with an active-site-directed inhibitor

**Fig. 2:**
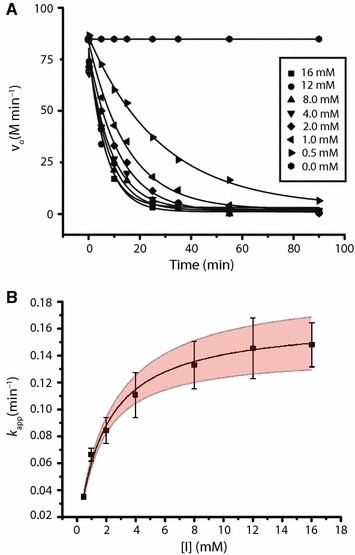
Inhibition kinetics of *Cj*GH5D with XXXG-NHCOCH_2_Br. **a** Initial-rate enzyme activity over time (single determinations). **b** Pseudo-first-order rate constants (*k*_app_) obtained from the fitted curves shown in **a**. Bars represent errors in *k*_app_ values from curve-fitting. The 95% confidence interval is indicated (pink band) for the fitted curve (solid line)

Activesite affinity-based inhibitors are important tools for the detailed kinetic analysis of GH enzymes [58]. In particular, *N*-bromoacetylglycosylamine derivatives of xyloglucan oligosaccharides have been previously demonstrated to be specific active-site affinity labels for *endo*-xyloglucanases [36, 59]. A time- and concentration-dependent inactivation of the enzyme *Cj*GH5D was observed upon incubation with XXXG-NHCOCH_2_Br, which followed pseudo-first-order kinetics (Fig. 2). The dissociation constant *K*_*i*_ and the irreversible inactivation constant *k*_*i*_ for XXXG-NHCOCH_2_Br towards *Cj*GH5D were 1.78 ± 0.17 mM and 0.17 ± 0.01 min^−1^, respectively, resulting in a *k*_i_/*K*_i_ value (9.3 × 10^−2^ mM^−1^ min^−1^) that was comparable to that previously observed for a *Prevotella bryantii* GH5_4 member (*Pb*GH5A) [36]. Notably, intact protein mass spectrometry of *Cj*GH5D following incubation with the inhibitor indicated covalent labelling with 1:1 stoichiometry and no over-labelling of the enzyme (Additional file 1: Figure S10).

### *Cj*GH5D crystallography

**Fig. 3:**
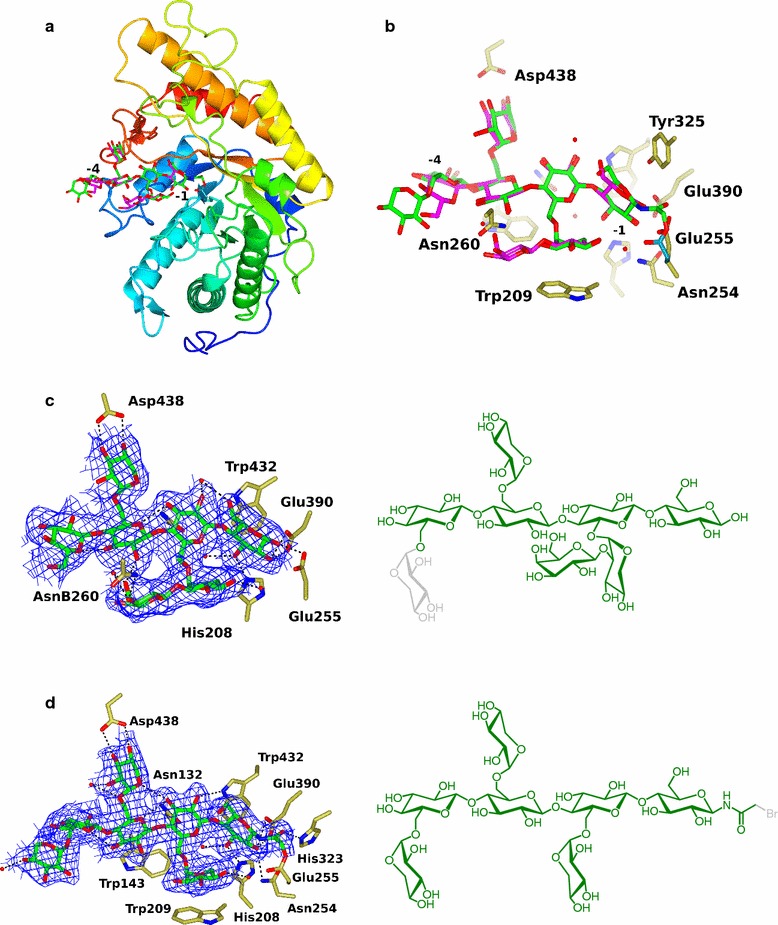
Three-dimensional structure of *Cj*GH5D in complex with XXXG-NHCOCH_2_Br and XyGOs. **a** Cartoon representation of the secondary structure of *Cj*GH5D colour ramped from the N-terminus (blue) to the C-terminus (red). The two ligands XXXG-NHCOCH_2_Br and GXLG are overlaid in the active site cleft and shown in green and magenta sticks, respectively. **b** A close-up view of the active site cleft with the overlaid ligands XXXG-NHCOCH_2_Br in green and XXLG in magenta showing different amino acids interacting with the carbohydrate ligands. **c** 2*F*_o_ − *F*_c_ (*σ*_A_/maximum likelihood weighted) electron density contoured in blue around GXLG in the *Cj*GH5D-XXLG complex (left panel) and the chemical structure of the corresponding ligand (Right panel). Insufficient electron density was observed for the − 4′ xylosyl residue to allow modelling, therefore, it is shown in grey. **d** 2*F*_o_ − *F*_c_ electron density at 1σ (approx. 0.2 *e*^−^/Å^3^) contoured in blue around XXXG-NHCOCH_2_ moiety in the *Cj*GH5D-XXXG-NHCOCH_2_Br complex (left panel) and chemical structure of the corresponding ligand (right panel). The bromide leaving group is shown in grey

A tertiary structure of the catalytic domain of *Cj*GH5D was determined at 1.6 Å resolution in uncomplexed “apo” form by X-ray crystallography and molecular replacement with *Bo*GH5A (pdb: 3ZMR, [22]). The overall structure of *Cj*GH5D (residues Gly96 to Gln468) is an (β/α)_8_ barrel as is typical for GH5 family members (Fig. 3a). Despite sequence identities in the 25–40% range, the structure is similar to the catalytic domains of many GH5 enzymes, most of which are annotated as xyloglucanases, glucanases and lichenases, with typical alignment values of approximately 310 residues aligning with an rmsd of 1.3 Å [60]. For example, the structure used for molecular replacement, *Bo*GH5A, overlaps with an r.m.s.d. of 1.1 Å over 332 equivalent Cα atoms with 40% identity. Minor differences are observed between the two structures in loops at the end of core helices. *Bo*GH5A has an extra loop Val170–Gly180 (residues equivalent to Ile137–Gly138 in *Cj*GH5D) which enables the formation of a hydrogen bond to the − 4′-xylosyl residue of ligand XXXG (between N Val182 and the sugar ring O atom, vide infra).

A 1.9 Å-resolution product complex of *Cj*GH5D was obtained by soaking crystals with a mixture of Glc_12_-based XyGOs of variable sidechain galactosylation. Here, we anticipated that the substrate mixture would be hydrolyzed and that the enzyme would selectively bind the oligosaccharide for which it had the best affinity. Commensurate with limit-digest analysis, we observed a Glc_4_-based oligosaccharide backbone spanning the − 4 to − 1 subsites for both molecules in the asymmetric unit: GXLG in molecule A (with glucose in the − 4 subsite and the − 3′-xylosyl group modelled at occupancies of 0.5 and 0.7, respectively) and GXXG in molecule B (here, there was insufficient electron density in the *F*_o_ − *F*_c_ difference map to allow unambiguous modelling of a galactose on the − 2′-xylosyl unit). In the − 1 subsite, the glucosyl residue interacts with the catalytic acid base Glu255 (via O1), and nucleophile Glu390 via O2. In addition, O3 is hydrogen bonded to His208. In molecule B, the equivalent glucose also hydrogen bonds via O2 to Asn254 and His208 (Fig. 3b, c).

**Fig. 4:**
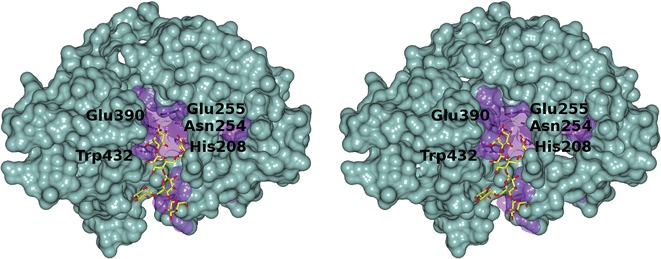
Divergent (wall-eyed) stereo surface representation of *Cj*GH5D-GXLG showing regions of sequence conservation. Surfaces of conserved and non-conserved residues, shown in purple at reduced opacity and sea-green, respectively, were calculated from an amino acid sequence alignment of GH5 domains of *Cj*GH5D, *Cj*GH5E, *Cj*GH5F and five additional GH5 members showing E.C. 3.2.1.151 activity (Additional file 1: Figure S4). Figure was generated using CCP4MG [81]

A second oligosaccharide complex was obtained at 2.1 Å resolution by soaking *Cj*GH5D crystals with the *N*-bromoacetyl affinity label XXXG-NHCOCH_2_Br, in which the reagent had indeed reacted through attack of the catalytic general acid/base sidechain to displace the bromide nucleofuge. In molecule A of the asymmetric unit, there is electron density for GXXG-NHCOCH_2_-*Cj*GH5D, whilst in molecule B, XXXG-NHCOCH_2_-*Cj*GH5D is modeled, but with the − 3′- and − 4′-xylosyl sugars modelled at half occupancy. The carboxyl oxygen of the *N*-acetyl moiety forms a hydrogen bond with His323. There are hydrogen bonds between this subsite − 1 sugar and the catalytic nucleophile Glu390, and also to His208 and Asn254 (Fig. 3b, d). These are similar to interactions observed in the structure of an analogous XXXG-NHCOCH_2_-*Pb*GH5A complex structure (pdb: 5D9P, [36]). Glucose in the − 2 subsite is hydrogen bonded via O3 to ND2 Asn132 and via O2 to NE1 Trp432; this latter interaction is notably long (ca. 3.2 Å), which may reflect the positioning of the tryptophan as the − 1 subsite stacking residue. The equivalent Asn/Trp interactions are also seen in related enzymes: Asn28 and Trp324 in the XXXG-NHCOCH_2_-*Pb*GH5A complex (pdb: 5D9P) and Asn165 and Trp472 in the *Bo*GH5A-XXXG complex (PDB 3ZMR). In addition to Trp432, Trp143 provides aromatic stacking interactions with the glucose in the − 3 subsite (homologous to Trp324 and Trp48 in *Pb*GH5A, and Trp472 and Trp185 in *Bo*GH5A, respectively), while Trp209 lies against the − 2′-xylosyl residue (as does the equivalent Trp252 in *Bo*GH5A). This pattern of conserved/highly invariant residues interacting with the xyloglucan chain presumably accounts for the fact that despite sharing amino acid identity as low as 30%, these enzymes are all tailored for xyloglucan as a substrate (Fig. 4). None of these three GH5D structures exhibits direct interactions with glucosyl units in the − 3 and − 4 subsites with the protein. The − 3′-xylosyl unit is tethered by two hydrogen bonds between O3 and O4 and Asp438, which hold the sugar perpendicular to the orientation of the equivalent xylose in the XXXG-NHCOCH_2_-*Pb*GH5A and *Bo*GH5A:XXXG complexes (in the latter, the xylose lies parallel to the side chain of Tyr476).

The covalent adduct formation through the reactivity of the *N*-bromoacetyl reagent is fascinating given that in the structures observed here, the attack is made by the acid–base Glu255, as opposed to the enzymatic nucleophile of the enzymatic reaction, Glu390. This latter residue is poised for nucleophilic attack at the anomeric carbon, C1, of the − 1 subsite glucoside. However, Glu390 is too distant (6–7 Å) from the reactive carbon of the *N*-bromoacetyl moiety, and has impossible geometry and steric hindrance, to permit nucleophilic interception. The reactive group, however, is located in the + 1 subsite—some 3.8 Å from C1—thus can be fortuitously attacked by the acid/base which is in almost ideal position for *S*_N_2 attack on the reactive carbon to displace the bromide. Such a reaction is facilitated, either prior, or subsequent to attack by rotation around the CB–CG bond, which leaves the side-chain in a different rotamer after the reaction relative to its “normal” position in unreacted complexes (Fig. 3d).

### Mutational analyses of *C. japonicus* GH5_4 genes indicate a complex mode of action for the initial stages of xyloglucan degradation

Facilitated by knowledge of their broadly similar catalytic properties, we embarked on a comprehensive reverse-genetic analysis in an attempt to delineate the biological functions of the individual GH5_4 and GH74 *endo*-xyloglucanases, using recently developed in-frame gene deletion techniques [9].

**Fig. 5:**
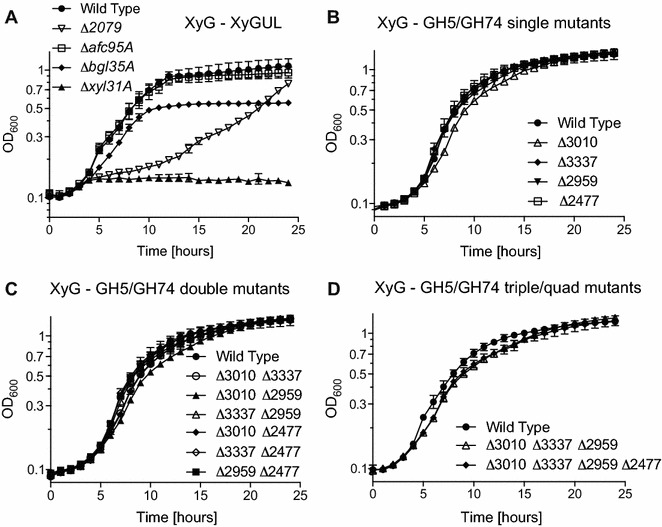
Growth analysis of in-frame deletions of GH5_4, and GH74 mutant strains on xyloglucan. **a** Control experiment with XyGUL in-frame deletion mutant strains. **b** Single, **c** double, **d** triple and quadruple deletion mutants were made with the GH5_4 and GH74 genes; CJA_3010 encodes *Cj*GH5D, CJA_3337 encodes *Cj*GH5E, CJA_2959 encodes *Cj*GH5F, and CJA_2477 encodes *Cj*GH74. Graphs represent the average of three biological replicates and error bars represent the standard deviation. All strains grew similarly to wild-type when grown with MOPS-glucose defined medium (Additional file 1: Figure S12)

In-frame deletion mutants were first generated in the XyG gene cluster encoding the three *exo*-glycosidases and the TBDT (Fig. 1b) to provide benchmark controls for subsequent analysis of *endo*-xyloglucanase deletion mutants. Recapitulating our previous work using insertional mutants [13], an in-frame Δ*xyl31A* (α-xylosidase) mutant was unable to grow on XyG due to an inability to remove non-reducing terminal xylosyl residues as the first essential step in XyGO saccharification (Fig. 5a, cf. Fig. 1). A ΔCJA_2709 (TBDT) single mutant strain had a significant growth defect, presumably resulting from a decreased ability to uptake extracellularly produced XyGOs into the periplasm. The deletion of *bgl35A* also attenuated growth, due to an inability of the strain to access the full complement of sidechain monosaccharides. As expected, growth of the Δ*afc95A* (α-l-fucosidase) mutant on tamarind (galacto)xyloglucan was identical to the wild-type strain, because this readily available substrate lacks the terminal fucosyl residues typically found in dicot primary cell wall XyG (Fig. 1a). Moreover, all XyG gene cluster mutant strains grew similarly to wild type in glucose containing medium (Additional file 1: Figure S11).

With these control experiments complete, we next analyzed the effect of deleting the individual GH5_4- and GH74-encoding genes. Despite original indications by RNAseq analysis of a potential lead role for *Cj*GH5D in XyG utilization, in-frame deletion of CJA_3010 surprisingly did not elicit a statistically significant growth defect (Fig. 5b). Likewise, strains containing single in-frame deletions of CJA_3337, CJA_2959, and CJA_2477 grew identically to the wild-type strain. Moreover, comprehensive combinatorial mutagenesis did not yield a strain with a substantial growth defect for any combination of double, triple, or quadruple mutants (Fig. 5c, d). Furthermore, the growth traits (maximum OD and growth rate) of both the wild-type and the quadruple deletion mutant were similar when reduced (0.25%) or limiting (0.125%) concentrations of XyG were used (Additional file 1: Figure S12 and Table S6). Collectively, these results suggest that despite their individual high activities and specificities toward XyG, as defined by the biochemical and structural analyses described above, the GH5_4 and GH74 members are not the only enzymes encoded by *C. japonicus* with sufficient xyloglucanase activity to support growth.

*Cj*GH5D is predicted to be attached to the exterior face of the outer membrane by N-terminal lipidation, while *Cj*GH5E, *Cj*GH5F, and *Cj*GH74 are likely to be secreted enzymes (vide supra). Thus, we hypothesized that other secreted enzymes, with either predominant or side hydrolytic activities toward XyG, may be enabling growth of the quadruple mutant. Deletion of the type-two secretion system (T2SS) in the Δ*gsp* mutant has been previously shown to abolish the ability of *C. japonicus* to secrete cellulases [11], and constitutes a powerful tool to restrict extracellular secretion of CAZymes in general. Interestingly, the introduction of the CJA_3010 deletion into the Δ*gsp* background resulted in only limited growth attenuation on xyloglucan (Additional file 1: Figure S13). With the T2SS extracellular secretion pathway disabled, the ability of the Δ*gsp* ΔCJA_3010 strain to grow on XyG strongly suggests the presence of other membrane-bound XGases that effect XyG depolymerization in a physiologically relevant manner. These data sharply contrast observations for the human gut symbiont *Bacteroides ovatus*, for which deletion of a single GH5_4 member from the XyGUL resulted in complete loss of growth on XyG [22].

Predominant xyloglucanase activity has been demonstrated previously in members of CAZyme families GH5, GH7, GH9, GH12, GH16, GH44, and GH74, and potentially may constitute a side activity in other *endo*-β(1,4)glucanases (cellulases) [23, 61]. Examination of the *C. japonicus* genome indicates the presence of multiple GH5 (*n* = 15), GH9 (*n* = 3), and GH16 (*n* = 9) encoding genes, in addition to the single GH74 member ([8]; for a summary table, see http://www.cazy.org/b776.html). Further, Deboy et al. [8] predicted that there are approximately 45 membrane-bound CAZymes. Although it constitutes a significant undertaking that is beyond the scope of the present study, our future investigations will focus on scrutinizing these additional CAZymes in the context of XyG utilization by *C. japonicus*.

## Conclusions

We previously proposed a model of XyG utilization by *C. japonicus*, in which an extracellular *endo*-xyloglucanase mediates degradation of the polysaccharide to XyGOs for uptake via the TBDT, followed by complete hydrolysis to monosaccharides in the periplasm by the *exo*-glycosidases encoded by the XyG gene cluster [13]. Our present study, combining biochemical and reverse-genetic analyses, reveals that the number of actors in the initial cleavage event is significantly greater than what was originally anticipated by bioinformatics. We propose that the existence of so many extracellular *endo*-xyloglucanases of apparently overlapping biochemical function can be explained by a physiological interplay of secreted reconnaissance enzymes and cell-surface-bound, proximal XyG degraders (Fig. 1b).

Thus, the secreted GH74 and two secreted GH5_4 enzymes may act as highly mobile, primary “unravellers” of the plant cell, liberating XyG fragments from the lignocellulose matrix (Fig. 1c). Indeed, the concept of “sensing” polysaccharidases playing a lead role in generating inducers has been previously proposed [62]. As plant cell wall polysaccharide degradation advances, more intimate contact between the bacterial cell surface and the substrate may ensue, engaging the outer-membrane-bound *Cj*GH5D and a more efficient interplay between XyG backbone hydrolysis and direct TBDT-mediated uptake of the oligosaccharide products. The coordinated capture, hydrolysis, and uptake of partially hydrolyzed polysaccharides as a successful competitive strategy has considerable precedent in the Polysaccharide Utilization Loci of the Bacteroidetes [63]. Moreover, the need to initiate cell wall “unravelling” has been suggested to explain why saprophytes such as *C. thermocellum*, which are unable to utilize xyloglucan or xylan for growth, contain *endo*-xyloglucanases and *endo*-xylanases within their cellulosomes [39, 64].

## Methods

### Transcriptomic analysis

RNAseq sampling and analysis was performed as previously described [9, 12]. Briefly, *C. japonicus* cultures were grown in 500 mL flasks at 30 °C shaking at 200 RPM. OD_600_ was measured every hour to monitor growth and samples were taken during exponential and stationary phase. Within 2 min of sampling, metabolism was stopped using a phenol/ethanol solution (5%/95%). The samples were immediately pelleted by centrifugation at 8000 *g* at 4 °C for 5 min. The supernatant was discarded and cell pellets were then flash frozen using a dry ice/ethanol bath and stored at − 80 °C. RNA extraction, library preparation, and sequencing was performed by GeneWIZ (South Plainfield, NJ). Illumina HiSeq 2500 was performed as 50 bp single-reads with at least 10 million reads generated per sample. The raw data have been submitted to the NCBI Gene Expression Omnibus (Accession GSE109594).

### Bioinformatic analysis

The full-length proteins encoded by ORFs CJA_3010 (*Cj*GH5D), CJA_3337 (*Cj*CBM2-CBM10-GH5E) and CJA_2959 (*Cj*FN3-GH5F) in *C. japonicus* genome were screened for the presence of a signal peptide using SignalP 4.0 [32] and LipoP 1.0 [65]. The modular architecture of the three enzymes was obtained from BLASTP analysis and additional alignment with representative GH and CBM modules from the CAZy Database [7] using ClustalW [66].

### Cloning of cDNA encoding protein modules

cDNA encoding the full-length enzymes *Cj*SRL-GH5D, *Cj*CBM2-CBM10-GH5E and *Cj*FN3-GH5F, in addition to the catalytic domains *Cj*GH5D, *Cj*GH5E and *Cj*GH5F were PCR amplified from *C. japonicus* genomic DNA; all constructs were designed such that the native predicted signal peptide was removed (PCR primers are listed in Additional file 1: Table S7). The amplified *Cj*SRL-GH5D, *Cj*CBM2-CBM10-GH5E, *Cj*FN3-GH5F, *Cj*GH5D and *Cj*GH5F products were double-digested with *Nhe*I and *Xho*I, gel purified and ligated to the respective sites of *pET28a* to fuse an N-terminal 6× His-Tag. The amplified *Cj*GH5E product was ligated in an SspI linearized pMCSG53 vector using the Ligation Independent Cloning (LIC) strategy [67]. Successful cloning was confirmed by PCR and plasmid DNA sequencing. Q5 high fidelity DNA polymerase was used for all the PCR amplifications.

### Gene expression and protein purification

Constructs were individually transformed into the chemically competent *E. coli* Rosetta DE3 cells. Colonies were grown on LB solid media containing kanamycin (50 µg mL^−1^) and chloramphenicol (30 µg mL^−1^) [*Cj*SRL-GH5D, *Cj*CBM2-CBM10-GH5E, *Cj*FN3-GH5F, *Cj*GH5D and *Cj*GH5F], or containing ampicillin (50 µg mL^−1^) and chloramphenicol (30 µg mL^−1^) [*Cj*GH5E]. One colony of the transformed *E. coli* cells was inoculated in 5 mL of LB medium containing the same antibiotics and grown overnight at 37 °C (200 rpm). The whole overnight culture was used to inoculate 500 mL of TB liquid medium containing the appropriate antibiotics. Cultures were grown at 37 °C (200 rpm) until *D*_600_ = 0.6. Overexpression was induced by adding IPTG to a final concentration of 0.1 mM. After induction, cultures were grown overnight at 16 °C (200 rpm). Cultures were then centrifuged (4220 *g* at 4 °C) and pellets were resuspended in 10 mL of *E. coli* lysis buffer containing 20 mM HEPES, pH 7.0, 500 mM NaCl, 40 mM imidazole, 5% glycerol, 1 mM DTT and 1 mM PMSF. Cells were then disrupted by sonication and the clear supernatant was separated by centrifugation at 4 °C (24,000 *g* for 45 min). Recombinant proteins were purified from the clear soluble lysates using a Ni^+2^-affinity column utilizing a gradient elution up to 100% elution buffer containing 20 mM HEPES, pH 7.0, 100 mM NaCl, 500 mM imidazole, and 5% glycerol in an FPLC system. Purity of the recombinant proteins was determined by visualizing the protein contents of the fractions on SDS-PAGE. Pure fractions were pooled, concentrated, and buffer exchanged against 50 mM phosphate buffer (pH 7.0) containing 10% glycerol. Protein concentrations were then determined using Epoch Micro-Volume Spectrophotometer System (BioTek^®^, USA) at 280 nm, and identities of the purified proteins were confirmed by intact mass spectrometry [68]. Purified proteins were then aliquoted and stored at − 80 °C until needed.

### Carbohydrate sources

Tamarind seed XyG, konjac glucomannan (KGM), barley β-glucan (BBG), wheat flour arabinoxylan, and beechwood xylan were purchased from Megazyme^®^ (Bray, Ireland). Hydroxyethylcellulose (HEC) was purchased from Amresco^®^ (Solon, USA). Carboxymethyl cellulose was purchased from Acros Organics (New Jersey, USA). Guar gum was purchased from Sigma Aldrich^®^ (St. Louise, USA). Xanthan gum was purchased from Spectrum^®^ (New Brunswick, USA). 2-Chloro-4-nitrophenyl (CNP)-β-d-cellotrioside (GGG-β-CNP) and CNP-β-d-cellotetraoside (GGGG-β-CNP) were purchased from Megazyme^®^. XXXG-β-CNP and XLLG-β-CNP were prepared as previously described [69]. Glc_4_-based XyGOs (XXXG, XLXG, XXLG, and XLLG; nomenclature according to [20]) and Glc_8_-based XyGOs were prepared from XyG powder (Innovassynth Technologies, Maharashtra, India) as previously described [61]. XXXG-NHCOCH_2_Br was synthesized as previously described [59].

### Carbohydrate analytics

High Performance Anion-Exchange Chromatography with Pulsed Amperometric Detection (HPAEC-PAD) was performed on a Dionex ICS-5000 DC HPLC system operated by the Chromeleon software version 7 (Dionex) using a Dionex Carbopac PA200 column. Solvent A was double-distilled water, solvent B was 1 M sodium hydroxide (NaOH), and solvent C was 1 M sodium acetate (NaOAc). The gradient used was: 0–4 min, 10% solvent B and 2.5% solvent C; 4–24 min, 10% B and a linear gradient from 2.5 to 25% C; 24–24.1 min, 50% B and 50% C; 24.1–25 min, an exponential gradient of NaOH and NaOAc back to initial conditions; and 25–31 min, initial conditions.

Matrix-assisted laser desorption ionization-time of flight (MALDI-TOF) was performed on a Bruker Daltonics Autoflex System (Billerica, USA). The matrix, 2,5-dihydroxy benzoic acid, was dissolved in 50% methanol in water to a final concentration of 10 mg mL^−1^. Oligosaccharide samples were mixed 1:1 (v/v) with the matrix solution. One microliter of this solution was placed on a Bruker MTP 384 ground steel MALDI plate and left to air dry for 2 h prior to analysis.

### Enzyme kinetic analysis

All enzyme activities toward polysaccharides were determined using a bicinchoninic acid (BCA) reducing-sugar assay [70]. The effect of temperature on xyloglucanase activity was determined by incubating the recombinant catalytic domain: *Cj*GH5D (0.098 µg), *Cj*GH5E (0.086 µg), *Cj*GH5F (0.017 µg) with tamarind seed xyloglucan at a final concentration of 1 mg mL^−1^. Citrate buffer (pH 6, *Cj*GH5D and *Cj*GH5F) or phosphate buffer (pH 7.5, *Cj*GH5E) was used to a final concentration of 50 mM in a total reaction volume of 200 µL. Reaction mixtures were incubated for 10 min at temperatures ranging from 25 to 80 °C prior to the BCA assay. To determine the pH-rate profile, the same XyG concentration was incubated with the same enzyme amounts, except for *Cj*GH5D (0.049 µg), for 10 min at 50 °C (*Cj*GH5D, *Cj*GH5F), or 55 °C (*Cj*GH5E), with 50 mM final concentration of the following buffers: citrate (pH 3–6.5), phosphate (pH 6.5–8), and glycine (pH 8.5–9).

For qualitative activity assessment against the other polysaccharide substrates, 1 µg of each recombinant enzyme was added to XyG, HEC, CMC, BBG, KGM, wheat flour arabinoxylan, beechwood xylan, xanthan gum, and guar gum to a final concentration of 2 mg mL^−1^ in 200 µL reaction volumes containing 50 mM phosphate buffer (pH 7.5: *Cj*GH5D and *Cj*GH5E, or pH 7: *Cj*GH5F). Mixtures were then incubated at 50 °C (*Cj*GH5D and *Cj*GH5F) or 55 °C (*Cj*GH5E) for 10 min before the generated reducing ends were detected using BCA assay.

To determine specific activity values of *Cj*GH5 enzymes toward XyG, final concentration of 0.75, 2.59, and 0.71 nM of the recombinant purified catalytic modules *Cj*GH5D, *Cj*GH5E, and *Cj*GH5F, respectively, was incubated with tamarind seed XyG (1 mg mL^−1^) in 200 µL reaction mixtures containing 50 mM phosphate buffer (pH 7.5: *Cj*GH5D and *Cj*GH5E, or pH 7: *Cj*GH5F). Likewise, specific activity values of *Cj*GH5 enzymes toward HEC were obtained by incubating *Cj*GH5E and *Cj*GH5F at a final concentration of 1.04 and 0.65 µM, respectively, with 2 mg mL^−1^ HEC in 200 µL reaction mixtures containing 50 mM phosphate buffer (pH 7.5: *Cj*GH5E or pH 7: *Cj*GH5F). For specific activity toward CMC, final concentration of 1.04 µM of the purified catalytic module *Cj*GH5E was incubated with CMC (2 mg mL^−1^) in 200 µL reaction volume containing 50 mM phosphate buffer (pH 7.5). All reaction mixtures were incubated at 50 °C (*Cj*GH5D and *Cj*GH5F) or 55 °C (*Cj*GH5E) for 10 min prior to the BCA assay and all assays were performed in triplicates.

To determine Michaelis–Menten parameters for XyG, eight different concentrations of XyG solutions were used over the range 0.025–1 mg mL^−1^. The recombinant enzymes *Cj*GH5D (0.007 µg), *Cj*GH5E (0.022 µg), and *Cj*GH5F (0.006 µg) were individually incubated with each XyG concentration at 50 °C (*Cj*GH5D and *Cj*GH5F) or 55 °C (*Cj*GH5E) for 10 min in a 200 µL final reaction mixture containing 50 mM phosphate buffer (pH 7.5: *Cj*GH5D and *Cj*GH5E, or pH 7: *Cj*GH5F). *K*_*m*_ and *k*_cat_ values were determined by non-linear fitting of the Michaelis–Menten equation to the data in Sigmaplot^®^ (Systat software Inc.)

To identify Michaelis–Menten constants for the chromogenic substrates, different dilution series were established to give final concentration ranges of 0.0625–8 mM (GGG-β-CNP), 0.0625–8 mM (GGGG-β-CNP), 0.002–4 mM (XXXG-β-CNP), and 0.002–2 mM (XLLG-β-CNP). Substrate mixtures (225 µL) containing 50 mM phosphate buffer with the optimum pH of the enzyme were pre-incubated for 10 min at the optimum temperature of the enzyme (vide supra). 25 µL of 10× *Cj*GH5D (to give 30–3800 nM final concentration according to the tested substrate), *Cj*GH5E (3–260 nM), and *Cj*GH5F (4- 650 nM) was added to the substrate mixtures before the release of the aglycone was continuously monitored by measuring the change in absorbance at 405 nM for 2 min in a Cary50 UV–visible spectrophotometer (Varian). CNP molar extinction coefficients were determined to be 17,288 M^−1^ cm^−1^ in 50 mM phosphate buffer pH 7 and 17,741 M^−1^ cm^−1^ in 50 mM phosphate buffer pH 7.5.

### Enzyme product analysis

To determine the limit-digest products of the *Cj*GH5 enzymes, 5 µg of each recombinant enzyme was incubated with tamarind seed XyG at final concentration of 0.25 mg mL^−1^ for 7 h (40 °C) in a 200 µL reaction mixture that contained 50 mM phosphate buffer of the optimum pH of the tested enzyme (pH 7.5: *Cj*GH5D and *Cj*GH5E, or pH 7: *Cj*GH5F). The reaction mixture was then diluted 5 times prior to product analysis by HPAEC-PAD. To determine the mode of action of the enzyme, 0.01 µg of *Cj*GH5 enzymes was incubated at 40 °C with 1 mg mL^−1^ final concentration of tamarind seed XyG in 200 µL reaction volumes containing the same buffers used in the limit-digest analysis. The reaction was stopped at different time points by adding 100 µL of 1 M NH_4_OH. Reaction mixtures were then diluted 2 times with water prior to product analysis by HPAEC-PAD.

### Inhibition kinetics and active-site labelling

Inhibition kinetic parameters were determined as previously described [59]. Briefly, a final concentration of 0.23 μM of *Cj*GH5D was incubated with a series of different concentrations (0.5–16 mM) of XXXG-NHCOCH_2_Br at 40 **°**C in 20 mM phosphate buffer (pH 7.5) for up to 90 min. BSA to a final concentration of 0.1 mg mL^−1^ was added to the inhibition mix to prevent the non-specific loss of activity. Small samples (10 µL) of the incubate were periodically diluted 1:100 in 20 mM phosphate buffer (pH 7.5), and 100 µL of the diluted incubate was added to 100 µL of the pre-incubated substrate XXXG-CNP at 40 °C (0.1 mM final substrate concentration in the assay). Residual activities of the enzyme were determined by measuring the rate of the release of the chromophore 2-chloro-4-nitrophenolate [69] at 405 nm in Agilent Cary 60 UV–vis Spectrophotometer. Initial-rate kinetics were measured in the strictly linear range of the enzyme. Equations 1 and 2 were used to determine *K*_i_ and *k*_i_ values by non-linear regression curve-fitting using OriginPro 2015 software as previously described [59].

1 $$V = V_{0} { \exp }\left( { - k_{\text{app}} t} \right) + y_{\text{offset}}$$

2 $$k_{\text{app}} = \frac{{k_{\text{i}} \left[ I \right]}}{{K_{\text{i}} + \left[ I \right]}}$$

Intact protein masses were determined on a Waters Xevo Q-TOF with a nanoACQUITY UPLC system, according to the method previously published [68], with 2.5 mM inhibitor and 4.52 μM of enzyme.

### Construction of *C. japonicus* mutants and growth conditions

In-frame deletion mutants were made as previously described [9]. Briefly, 500 bp regions up- and down-stream of the genes of interest were amplified by PCR (CJA_3010, CJA_3337, and CJA_2959) or synthesized by GeneWIZ (South Plainfield, NJ) (CJA_2477) and assembled into pK18*mobsacB* by the method of Gibson et al. [71]. Deletions were confirmed by PCR. For a complete list of primers used see Additional file 1: Table S8. Cultures were grown at 30 °C with 200 RPM shaking in MOPS minimal media containing 0.25% (w:v) glucose, 0.25%  (w:v) XyG or 0.5% (w:v) XyG as the sole carbon source in 18 mm test tubes or in 96 well flat bottom polystyrene plates (Corning). Growth was measured using a Spec20D+ spectrophotometer (Thermo Scientific) or a Tecan Plate reader (Tecan, Switzerland). All experiments were performed in biological triplicate. Statistical analysis was performed using Graphpad Prism 6 software package (La Jolla, CA) where appropriate.

### Crystallization, X-ray crystallography and structure solution

*Cj*GH5D was crystallized in sitting drops by the Vapour Diffusion method, using protein at 21 mg mL^−1^ in 50 mM sodium citrate pH 6.5 and 10% glycerol over a well solution comprised of 1.9 M ammonium sulfate, 0.1 M 2-(*N*-morpholino)ethanesulfonic acid (MES) pH 5.5. The drop consisted of 0.5 μL enzyme, 0.1 μL seed stock and 0.4 μL well solution, and the seed stock was prepared by vortexing crystals, grown over 1.4 M ammonium sulfate, 0.1 M MES pH 5.5, 1% (w/v) polyethylene glycol 1000, in an Eppendorf tube with a polystyrene bead. Crystals were harvested into liquid nitrogen using nylon CryoLoops™ (Hampton Research). A non-ligand complexed “apo” dataset was collected from a crystal after immersing for a few minutes in a cryoprotectant solution comprised of the mother liquor supplemented with 20% (v/v) glycerol. Data were collected at Diamond beamline I04, and processed using *DIALS* [72], and scaled using *AIMLESS* [73] to 1.6 Å. The space group is P2_1_2_1_2_1_ with unit cell dimensions 55.0, 96.4, 159.0 Å, and there are 2 molecules in the asymmetric unit.

The structure was solved by molecular replacement using *Phaser* [74] using residues 138–500 of PDB entry 3zmr as the search model [22], which align to residues 106–464 of *Cj*GH5D, with which they share 38% identity (using the program *lalign* from the FASTA package [75]). The structure was built automatically using *Buccaneer* [76] and refined using cycles of manual model rebuilding using *Coot* [77] followed by refinement with *REFMAC* [78], including cycles using anisotropic B-factor refinement. In addition to the two protein chains, there are 20 molecules of glycerol, 5 sulfate ions and 3 molecules of PEG (introduced from the seed stock solution). Data collection and refinement statistics for all structures are given in Additional file 1: Table S9.

A crystal of *Cj*GH5D, grown as above over a well solution comprised of 2.3 M ammonium sulfate, 0.1 M MES pH 5.5, was soaked for 27.5 h in 1.85 M ammonium sulfate, 0.1 M MES pH 5.5 with 4.5 mM XXXG-NHCOCH_2_Br, and fished directly into liquid nitrogen. Data were collected at Diamond beamline I03, and processed using *DIALS* [72]. After scaling with *AIMLESS* [73], the data were cut off at a resolution of 2.1 Å, as although the X-ray images showed significant spot smearing, the *R*_merge_ and CC_1/2_ values were good (6.2% overall, 54.7% in the outer shell and 0.998 overall, 0.933 in the outer shell, respectively).

Crystals grown under similar conditions, over a well containing 1.6 M ammonium sulfate, 0.1 M MES pH 5.5, were soaked in the presence of a mixture of Glc_12_-based XyGOs (produced as described in [46] at a concentration of 5 mM for 5 h, before fishing into liquid nitrogen via a cryoprotectant solution, as for the apo crystal. A dataset was collected at Diamond beamline I04 and processed using *DIALS* and scaled using *AIMLESS* to 1.9 Å.

Both ligand structures were solved initially using the apo structure as a model for *REFMAC*, and the ligand was placed after the protein chain had been rebuilt (using cycles of Coot interspersed with refinement in *REFMAC*) and some water molecules added. All models were validated using *MolProbity* [79] and the sugar conformations of the ligand in the complex structures were checked using Privateer [80]. Problems with diffraction anisotropy in both ligand datasets limited the possibility of refining the structures to R/Rfree lower than 0.22/0.28 and 0.23/0.30 for the complexes with XXXG-NHCOCH_2_ and GXLG (produced after the Glc_12_ soak), respectively.

## Additional file

**Additional file 1.** Supplemental Tables S1–S9 and Supplemental Figures S1–S13.

## Abbreviations

XyG: xyloglucan
XyGO: xylogluco-oligosaccharides
CAZy: carbohydrate active enzymes
GH5: glycoside hydrolase family 5
CBM: carbohydrate binding module
SRL: serine rich linker
HPAEC-PAD: high performance anion exchange chromatography-pulsed amperometric detector
MALDI-TOF: matrix assisted laser desorption ionization-time of flight
KGM: konjac glucomannan
BBG: barley β-glucan
HEC: hydroxyethylcellulose
CNP: 2-chloro-4-nitrophenyl
BCA: bicinchoninic acid
TBDT: TonB-dependent transporter
XyGUL: xyloglucan utilization locus
MES: 0.1 M 2-(*N*-morpholino)ethanesulfonic acid

## References

[1] Parajuli R, Dalgaard T, Jorgensen U, Adamsen APS, Knudsen MT, Birkved M, Gylling M, Schjorring JK (2015). Biorefining in the prevailing energy and materials crisis: a review of sustainable pathways for biorefinery value chains and sustainability assessment methodologies. Renew Sustain Energy Rev.

[2] Tuck CO, Perez E, Horvath IT, Sheldon RA, Poliakoff M (2012). Valorization of biomass: deriving more value from waste. Science.

[3] Horn SJ, Vaaje-Kolstad G, Westereng B, Eijsink VGH (2012). Novel enzymes for the degradation of cellulose. Biotechnol Biofuels.

[4] Li LL, McCorkle SR, Monchy S, Taghavi S, van der Lelie D (2009). Bioprospecting metagenomes: glycosyl hydrolases for converting biomass. Biotechnol Biofuels.

[5] Gardner JG (2016). Polysaccharide degradation systems of the saprophytic bacterium *Cellvibrio japonicus*. World J Microbiol Biotechnol.

[6] Hazlewood GP, Gilbert HJ (1998). Structure and function analysis of *Pseudomonas* plant cell wall hydrolases. Biochem Soc Trans.

[7] Lombard V, Ramulu HG, Drula E, Coutinho PM, Henrissat B (2014). The carbohydrate-active enzymes database (CAZy) in 2013. Nucleic Acids Res.

[8] Deboy RT, Mongodin EF, Fouts DE, Tailford LE, Khouri H, Emerson JB, Mohamoud Y, Watkins K, Henrissat B, Gilbert HJ, Nelson KE (2008). Insights into plant cell wall degradation from the genome sequence of the soil bacterium *Cellvibrio japonicus*. J Bacteriol.

[9] Nelson CE, Gardner JG (2015). In-frame deletions allow functional characterization of complex cellulose degradation phenotypes in *Cellvibrio japonicus*. Appl Environ Microbiol.

[10] Gardner JG, Keating DH (2012). Genetic and functional genomic approaches for the study of plant cell wall degradation in *Cellvibrio japonicus*. Methods Enzymol.

[11] Gardner JG, Keating DH (2010). Requirement of the type II secretion system for utilization of cellulosic substrates by *Cellvibrio japonicus*. Appl Environ Microbiol.

[12] Gardner JG, Crouch L, Labourel A, Forsberg Z, Bukhman YV, Vaaje-Kolstad G, Gilbert HJ, Keating DH (2014). Systems biology defines the biological significance of redox-active proteins during cellulose degradation in an aerobic bacterium. Mol Microbiol.

[13] Larsbrink J, Thompson AJ, Lundqvist M, Gardner JG, Davies GJ, Brumer H (2014). A complex gene locus enables xyloglucan utilization in the model saprophyte *Cellvibrio japonicus*. Mol Microbiol.

[14] Carpita N, McCann M, Buchanan BB, Gruissem W, Jones RL (2000). The cell wall. Biochemistry and molecular biology of plants.

[15] Fangel JU, Ulvskov P, Knox JP, Mikkelsen MD, Harholt J, Popper ZA, Willats WGT (2012). Cell wall evolution and diversity. Front Plant Sci.

[16] Vogel J (2008). Unique aspects of the grass cell wall. Curr Opin Plant Biol.

[17] Scheller HV, Ulvskov P (2010). Hemicelluloses. Annu Rev Plant Biol.

[18] Hsieh YSY, Harris PJ (2009). Xyloglucans of monocotyledons have diverse structures. Mol Plant.

[19] Hoffman M, Jia ZH, Pena MJ, Cash M, Harper A, Blackburn AR, Darvill A, York WS (2005). Structural analysis of xyloglucans in the primary cell walls of plants in the subclass Asteridae. Carbohyd Res.

[20] Tuomivaara ST, Yaoi K, O’Neill MA, York WS (2015). Generation and structural validation of a library of diverse xyloglucan-derived oligosaccharides, including an update on xyloglucan nomenclature. Carbohyd Res.

[21] Pauly M, Keegstra K (2016). Biosynthesis of the plant cell wall matrix polysaccharide xyloglucan. Annu Rev Plant Biol.

[22] Larsbrink J, Rogers TE, Hemsworth GR, McKee LS, Tauzin AS, Spadiut O, Klinter S, Pudlo NA, Urs K, Koropatkin NM (2014). A discrete genetic locus confers xyloglucan metabolism in select human gut Bacteroidetes. Nature.

[23] Attia MA, Brumer H (2016). Recent structural insights into the enzymology of the ubiquitous plant cell wall glycan xyloglucan. Curr Opin Struct Biol.

[24] Larsbrink J, Izumi A, Ibatullin FM, Nakhai A, Gilbert HJ, Davies GJ, Brumer H (2011). Structural and enzymatic characterization of a glycoside hydrolase family 31 alpha-xylosidase from *Cellvibrio japonicus* involved in xyloglucan saccharification. Biochem J.

[25] Silipo A, Larsbrink J, Marchetti R, Lanzetta R, Brumer H, Molinaro A (2012). NMR spectroscopic analysis reveals extensive binding interactions of complex xyloglucan oligosaccharides with the *Cellvibrio japonicus* glycoside hydrolase family 31 *α*-xylosidase. Chem Eur J.

[26] Nelson CE, Attia MA, Rogowski A, Morland C, Brumer H, Gardner JG (2017). Comprehensive functional characterization of the glycoside hydrolase family 3 enzymes from *Cellvibrio japonicus* reveals unique metabolic roles in biomass saccharification. Environ Microbiol.

[27] Attia M, Stepper J, Davies GJ, Brumer H (2016). Functional and structural characterization of a potent GH74 *endo*-xyloglucanase from the soil saprophyte *Cellvibrio japonicus* unravels the first step of xyloglucan degradation. FEBS J.

[28] Aspeborg H, Coutinho PM, Wang Y, Brumer H, Henrissat B (2012). Evolution, substrate specificity and subfamily classification of glycoside hydrolase family 5 (GH5). BMC Evolut Biol.

[29] Raman B, McKeown CK, Rodriguez M, Brown SD, Mielenz JR (2011). Transcriptomic analysis of *Clostridium thermocellum* ATCC 27405 cellulose fermentation. BMC Microbiol.

[30] Riederer A, Takasuka TE, Makino S, Stevenson DM, Bukhman YV, Elsen NL, Fox BG (2011). Global gene expression patterns in *Clostridium thermocellum* as determined by microarray analysis of chemostat cultures on cellulose or cellobiose. Appl Environ Microbiol.

[31] Paetzel M, Karla A, Strynadka NCJ, Dalbey RE (2002). Signal peptidases. Chem Rev.

[32] Petersen TN, Brunak S, von Heijne G, Nielsen H (2011). SignalP 4.0: discriminating signal peptides from transmembrane regions. Nat Methods.

[33] Boraston AB, Bolam DN, Gilbert HJ, Davies GJ (2004). Carbohydrate-binding modules: fine-tuning polysaccharide recognition. Biochem J.

[34] Gloster TM, Ibatullin FM, Macauley K, Eklof JM, Roberts S, Turkenburg JP, Bjornvad ME, Jorgensen PL, Danielsen S, Johansen KS (2007). Characterization and three-dimensional structures of two distinct bacterial xyloglucanases from families GH5 and GH12. J Biol Chem.

[35] dos Santos CR, Cordeiro RL, Wong DWS, Murakami MT (2015). Structural basis for xyloglucan specificity and alpha-d-Xylp(1→6)-d-Glcp recognition at the − 1 subsite within the GH5 family. Biochemistry.

[36] McGregor N, Morar M, Fenger TH, Stogios P, Lenfant N, Yin V, Xu XH, Evdokimova E, Cui H, Henrissat B (2016). Structure-function analysis of a mixed-linkage beta-glucanase/xyloglucanase from the key ruminal bacteroidetes *Prevotella bryantii* B(1)4. J Biol Chem.

[37] Wong D, Chan VJ, McCormack AA, Batt SB (2010). A novel xyloglucan-specific *endo*-beta-1,4-glucanase: biochemical properties and inhibition studies. Appl Microbiol Biotechnol.

[38] Matsuzawa T, Saito Y, Yaoi K (2014). Key amino acid residues for the *endo*-processive activity of GH74 xyloglucanase. FEBS Lett.

[39] Ravachol J, Borne R, Tardif C, de Philip P, Fierobe H-P (2014). Characterization of all family-9 glycoside hydrolases synthesized by the cellulosome-producing bacterium *Clostridium cellulolyticum*. J Biol Chem.

[40] Ravachol J, de Philip P, Borne R, Mansuelle P, Maté MJ, Perret S, Fierobe HP (2016). Mechanisms involved in xyloglucan catabolism by the cellulosome-producing bacterium *Ruminiclostridium cellulolyticum*. Sci Rep.

[41] Song S, Tang YB, Yang SQ, Yan QJ, Zhou P, Jiang ZQ (2013). Characterization of two novel family 12 xyloglucanases from the thermophilic *Rhizomucor miehei*. Appl Microbiol Biotechnol.

[42] Damasio ARL, Ribeiro LFC, Ribeiro LF, Furtado GP, Segato F, Almeida FBR, Crivellari AC, Buckeridge MS, Souza TACB, Murakami MT (2012). Functional characterization and oligomerization of a recombinant xyloglucan-specific *endo*-beta-1,4-glucanase (GH12) from *Aspergillus niveus*. Biochimica Et Biophysica Acta-Proteins Proteomics.

[43] Master ER, Zheng Y, Storms R, Tsang A, Powlowski J (2008). A xyloglucan-specific family 12 glycosyl hydrolase from *Aspergillus niger*: recombinant expression, purification and characterization. Biochem J.

[44] Powlowski J, Mahajan S, Schapira M, Master ER (2009). Substrate recognition and hydrolysis by a fungal xyloglucan-specific family 12 hydrolase. Carbohyd Res.

[45] Eklof JM, Shojania S, Okon M, McIntosh LP, Brumer H (2013). Structure-function analysis of a broad specificity populus trichocarpa *endo*-beta-glucanase reveals an evolutionary link between bacterial licheninases and plant XTH gene products. J Biol Chem.

[46] Martinez-Fleites C, Guerreiro C, Baumann MJ, Taylor EJ, Prates JAM, Ferreira LMA, Fontes C, Brumer H, Davies GJ (2006). Crystal structures of *Clostridium thermocellum* xyloglucanase, XGH74A, reveal the structural basis for xyloglucan recognition and degradation. J Biol Chem.

[47] Yaoi K, Nakai T, Kameda Y, Hiyoshi A, Mitsuishi Y (2005). Cloning and characterization of two xyloglucanases from *Paenibacillus* sp. strain KM21. Appl Environ Microbiol.

[48] Yaoi K, Kondo H, Hiyoshi A, Noro N, Sugimoto H, Tsuda S, Miyazaki K (2009). The crystal structure of a xyloglucan-specific *endo*-beta-1,4-glucanase from *Geotrichum* sp. M128 xyloglucanase reveals a key amino acid residue for substrate specificity. FEBS J.

[49] Yaoi K, Mitsuishi Y (2004). Purification, characterization, cDNA cloning, and expression of a xyloglucan endoglucanase from *Geotrichum* sp. M128. FEBS Lett.

[50] Enkhbaatar B, Temuujin U, Lim JH, Chi WJ, Chang YK, Hong SK (2012). Identification and characterization of a xyloglucan-specific family 74 glycosyl hydrolase from *Streptomyces coelicolor A3*(2). Appl Environ Microbiol.

[51] Desmet T, Cantaert T, Gualfetti P, Nerinckx W, Gross L, Mitchinson C, Piens K (2007). An investigation of the substrate specificity of the xyloglucanase Cel74A from *Hypocrea jecorina*. FEBS J.

[52] Ariza A, Eklof JM, Spadiut O, Offen WA, Roberts SM, Besenmatter W, Friis EP, Skjot M, Wilson KS, Brumer H, Davies G (2011). Structure and activity of *Paenibacillus polymyxa* xyloglucanase from glycoside hydrolase family 44. J Biol Chem.

[53] Feng T, Yan K-P, Mikkelsen MD, Meyer AS, Schols HA, Westereng B, Mikkelsen JD (2014). Characterisation of a novel *endo*-xyloglucanase (XcXGHA) from *Xanthomonas* that accommodates a xylosyl-substituted glucose at subsite-1. Appl Microbiol Biotechnol.

[54] Yaoi K, Mitsuishi Y (2002). Purification, characterization, cloning, and expression of a novel xyloglucan-specific glycosidase, oligoxyloglucan reducing end-specific cellobiohydrolase. J Biol Chem.

[55] Ichinose H, Araki Y, Michikawa M, Harazono K, Yaoi K, Karita S, Kaneko S (2012). Characterization of an *endo*-processive-type xyloglucanase having a beta-1,4-glucan-binding module and an *endo*-type xyloglucanase from *Streptomyces avermitilis*. Appl Environ Microbiol.

[56] Davies GJ, Wilson KS, Henrissat B (1997). Nomenclature for sugar-binding subsites in glycosyl hydrolases. Biochem J.

[57] Barras F, Bortoligerman I, Bauzan M, Rouvier J, Gey C, Heyraud A, Henrissat B (1992). Stereochemistry of the hydrolysis reaction catalyzed by endoglucanase Z from *Erwinia chrysanthemi*. FEBS Lett.

[58] Gloster TM, Vocadlo DJ (2012). Developing inhibitors of glycan processing enzymes as tools for enabling glycobiology. Nat Chem Biol.

[59] Fenger TH, Brumer H (2015). Synthesis and analysis of specific covalent inhibitors of *endo*-xyloglucanases. ChemBioChem.

[60] Krissinel E, Henrick K (2004). Secondary-structure matching (SSM), a new tool for fast protein structure alignment in three dimensions. Acta Crystallogr Sect D Biol Crystallogr.

[61] Eklof JM, Ruda MC, Brumer H (2012). Distinguishing xyloglucanase activity in *endo*-beta(1→4)glucanases. Methods Enzymol.

[62] Zhao YX, Chany CJ, Sims PFG, Sinnott ML (1997). Definition of the substrate specificity of the ‘sensing’ xylanase of *Streptomyces cyaneus* using xylooligosaccharide and cellooligosaccharide glycosides of 3,4-dinitrophenol. J Biotechnol.

[63] Grondin JM, Tamura K, Déjean G, Abbott DW, Brumer H (2017). Polysaccharide utilization loci: fuelling microbial communities. J Bacteriol.

[64] Raman B, Pan C, Hurst GB, Rodriguez M, McKeown CK, Lankford PK, Samatova NF, Mielenz JR (2009). Impact of pretreated switchgrass and biomass carbohydrates on *Clostridium thermocellum* ATCC 27405 cellulosome composition: a quantitative proteomic analysis. PloS ONE.

[65] Rahman O, Cummings SP, Harrington DJ, Sutcliffe IC (2008). Methods for the bioinformatic identification of bacterial lipoproteins encoded in the genomes of Gram-positive bacteria. World J Microbiol Biotechnol.

[66] Thompson JD, Higgins DG, Gibson TJ (1994). CLUSTAL-W: improving the sensitivity of progressive multiple sequence alignment through sequence weighting, position-specific gap penalties and weight matrix choice. Nucleic Acids Res.

[67] Eschenfeldt WH, Lucy S, Millard CS, Joachimiak A, Mark ID (2009). A family of LIC vectors for high-throughput cloning and purification of proteins. Methods Mol Biol.

[68] Sundqvist G, Stenvall M, Berglund H, Ottosson J, Brumer H (2007). A general, robust method for the quality control of intact proteins using LC–ESI–MS. J Chromatogr B.

[69] Ibatullin FM, Baumann MJ, Greffe L, Brumer H (2008). Kinetic analyses of retaining *endo*-(xylo)glucanases from plant and microbial sources using new chromogenic xylogluco-oligosaccharide aryl glycosides. Biochemistry.

[70] McFeeters RF (1980). A manual method for reducing sugar determinations with 2,2′-bicinchoninate reagent. Anal Biochem.

[71] Gibson DG, Young L, Chuang RY, Venter JC, Hutchison CA, Smith HO (2009). Enzymatic assembly of DNA molecules up to several hundred kilobases. Nat Methods.

[72] Waterman DG, Winter G, Gildea RJ, Parkhurst JM, Brewster AS, Sauter NK, Evans G (2016). Diffraction-geometry refinement in the DIALS framework. Acta Crystallogr Sect D Struc Biol.

[73] Evans PR, Murshudov GN (2013). How good are my data and what is the resolution?. Acta Crystallogr Sect D.

[74] McCoy AJ, Grosse-Kunstleve RW, Adams PD, Winn MD, Storoni LC, Read RJ (2007). Phaser crystallographic software. J Appl University of YorkCrystallogr.

[75] Pearson WR, Stephen Misener SAK (1999). Flexible sequence similarity searching with the FASTA3 program package. Bioinformatics methods and protocols.

[76] Cowtan K (2006). The Buccaneer software for automated model building. 1. Tracing protein chains. Acta Crystallogr Sect D-Biol Crystallogr.

[77] Emsley P, Lohkamp B, Scott WG, Cowtan K (2010). Features and development of coot. Acta Crystallogr Sect D-Biol Crystallogr.

[78] Murshudov GN, Skubak P, Lebedev AA, Pannu NS, Steiner RA, Nicholls RA, Winn MD, Long F, Vagin AA (2011). REFMAC5 for the refinement of macromolecular crystal structures. Acta Crystallogr Sect D-Biol Crystallogr.

[79] Chen VB, Arendall WB, Headd JJ, Keedy DA, Immormino RM, Kapral GJ, Murray LW, Richardson JS, Richardson DC (2010). MolProbity: all-atom structure validation for macromolecular crystallography. Acta Crystallogr Sect D-Biol Crystallogr.

[80] Agirre J, Iglesias-Fernandez J, Rovira C, Davies GJ, Wilson KS, Cowtan KD (2015). Privateer: software for the conformational validation of carbohydrate structures. Nat Struct Mol Biol.

[81] McNicholas S, Potterton E, Wilson KS, Noble MEM (2011). Presenting your structures: the CCP4 mg molecular-graphics software. Acta Crystallogr Sect D-Biol Crystallogr.

