# Supplementary material for: In vitro and in vivo characterization of three Cellvibrio japonicus glycoside hydrolase family 5 members reveals potent xyloglucan backbone-cleaving functions
Source: Biotechnol Biofuels. 2018 Feb 17;11:45. doi: 10.1186/s13068-018-1039-6 (PMC5816542; doi:10.1186/s13068-018-1039-6)
Supplement: Supplementary file 1 — Additional file 1. Supplemental Tables S1–S9 and Supplemental Figures S1–S13. [file 13068_2018_1039_MOESM1_ESM.pdf]

Supporting Information: *In vitro* and *in vivo* characterization of three *Cellvibrio japonicus* Glycoside Hydrolase Family 5 members reveals potent xyloglucan backbone-cleaving functions

Mohamed A. Attia[1,2], Cassandra E. Nelson[3], Wendy A. Offen[4], Namrata Jain[1,2], Gideon J. Davies[4], Jeffrey G. Gardner[3], and Harry Brumer[1,2,5,6,*]

[1]Michael Smith Laboratories, University of British Columbia, 2185 East Mall, Vancouver, BC, V6T 1Z4, Canada.

[2]Department of Chemistry, University of British Columbia, 2036 Main Mall, Vancouver, British Columbia V6T 1Z1, Canada.

[3]Department of Biological Sciences, University of Maryland, Baltimore County, Baltimore MD 21250, USA.

[4]Department of Chemistry, University of York, Heslington, York YO10 5DD, UK.

[5]Department of Biochemistry and Molecular Biology, University of British Columbia, 2350 Health Sciences Mall, Vancouver, British Columbia V6T 1Z3, Canada.

[6]Department of Botany, University of British Columbia, 6270 University Blvd., Vancouver, British Columbia V6T 1Z4, Canada

*To whom correspondence should be addressed: E_mail brumer@msl.ubc.ca; Tel. (+1) 6048273738; Fax (+1) 6048222114.

Dedication: *This article is dedicated to Cellvibrio japonicus vanguard Prof. Harry J. Gilbert on the occasion of his retirement.*

Supplemental Tables

Table S1. List of genes up-regulated during exponential growth on xyloglucan compared to glucose[a].

Locus ID	Gene[b]	Predicted Function[b]	Fold Change[c]	p-value[d]
CJA_3010	*cel5D*	cellulase	4.9	5.0
CJA_2709	CJA_2709	TBD- transporter	4.4	4.8
CJA_2706	*xyl31A*	α-xylosidase[e]	4.2	4.0
CJA_0491	*gal53A-1*	arabinogalactan *endo*-1,4 β-galactosidase	4.2	3.2
CJA_3008	*xyl39A*	β-xylosidase	4.1	4.9
CJA_3007	*gly43H*	β-xylosidase/α-L-arabinfuranosidase[f]	4.0	5.0
CJA_2707	*bgl35A*	β-galactosidase[g]	3.6	3.0
CJA_2769	*abf51A*	α-L-arabinofuranosidase[h]	3.6	4.6
CJA_0496	*bgl2A*	β-galactosidase	3.5	3.5
CJA_1140	*cel3D*	cellodextrinase	3.1	3.7
CJA_2710	*afc95A*	α-L-fucosidase[g]	3.1	2.7
CJA_0492	*gal53B*	arabinogalactan *endo*-1,4-β-galactosidase	2.8	3.9
CJA_0497	*gal53A-2*	arabinogalactan *endo*-1,4-β-galactosidase[i]	2.5	2.9
CJA_0246	*aga27A*	α-galactosidase[j]	2.3	2.3
CJA_3018	*gly43N*	β-xylosidase/α-L-arabinfuranosidase[f]	1.9	2.1
CJA_0818	*gly43D*	β-xylosidase/α-L-arabinfuranosidase[f]	1.8	2.8
CJA_0181	*pme8C*	pectin methylesterase	1.7	2.5
CJA_0007	*cbp2A*	carbohydrate binding protein	1.6	4.5
CJA_0398	*amy13F*	α-amylase	1.6	2.1
CJA_3763	*xyn11A*	*endo*-1,4-β-xylanase[k]	1.6	2.1
CJA_0799	*gly43E*	β-xylosidase/α-L-arabinfuranosidase[f]	1.5	2.5
CJA_0817	*agd97A*	α-glucosidase	1.5	2.3
CJA_0223	*cel3C*	β-glucosidase	1.4	2.0
CJA_1182	*chi19A*	chitinase	1.3	2.1
CJA_2469	*cbp2F*	carbohydrate binding protein	1.3	2.3
CJA_0819	*abf43M*	α-L-arabinofuranosidase[f]	1.1	2.1
CJA_3120	*pel1G*	pectate lyase	1.1	2.1
CJA_2869	*cbp26A*	carbohydrate binding protein	1.1	3.4

[a] RNASeq sampling performed in biological triplicate
[b] Gene names and predicted functions according to Deboy *et al.* [1]
[c] $\log_2$ scale
[d] $-\log_{10}$ conversion
[e] Function confirmed by Larsbrink, *et al.* [2]
[f] Function confirmed by Cartmell, *et al.* [3]
[g] Function confirmed by Larsbrink, *et al.* [4]
[h] Function confirmed by Beylot, *et al.* [5]
[i] Function confirmed by Braithwaite, *et al.* [6]
[j] Function confirmed by Halstead, *et al.* [7]
[k] Function confirmed by Milward-Sadler, *et al.* [8]

Table S2. List of genes with low-level constitutive expression on xyloglucan[a].

Locus ID	Gene[b]	Predicted Function[b]	RPKM[c] (Exp)	RPKM (Sta)
CJA_0805	*arb43A*	α-L-arabinfuranosidase	102	130
CJA_3139	*lpmo10B*	lytic polysaccharide mono-oxygenase[d]	169	102
CJA_0276	*cbp6B*	carbohydrate binding protein	110	125
CJA_3300	*cbp6C*	carbohydrate binding protein	134	100
CJA_0374	*cel45A*	cellulase	157	153
CJA_0619	*cel5H*	*endo*-1,4 β-glucanase	100	108
CJA_3286	*ebg98*	*endo*-β-galactosidase	118	165
CJA_3287	*fee1A*	ferruloyl esterase	116	104
CJA_3282	*fee1B*	ferruloyl esterase	152	241
CJA_2477	*gly74A*	*endo*-1,4-β-glucanase/xyloglucanase	126	123
CJA_0384	*pel10C*	pectate lyase	139	115
CJA_2413	*pel3B*	pectate lyase	130	126
CJA_0172	*pga28A*	polygalacturonase	119	177
CJA_0284	*tre37B*	trehalase	178	136
CJA_3762	*xyn11B*	*endo*-1,4-β-xylanase	139	140

[a] RNASeq sampling performed in biological triplicate
[b] Gene names and predicted function according to Deboy *et al.*[1]
[c] Reads per kilobase per million mapped reads (average)
[d] Function confirmed by Gardner, *et al.* 2014 [9].

Table S3. List of genes up-regulated during stationary phase on xyloglucan compared to glucose[a].

Locus ID	Gene	Predicted Function[b]	Fold Change[c]	p-value[d]
CJA_2618	*amy13A*	α-amylase	3.0	3.3
CJA_0806	*abf43L*	α-L-arabinofuranosidase[e]	3.0	2.9
CJA_2611	*chi18D*	*endo*-chitanase	2.8	2.8
CJA_3247	*amy13H*	α-amylase	2.7	2.3
CJA_2710	*afc95A*	α-L-fucosidase[f]	2.6	2.3
CJA_0020	*cbp35A*	carbohydrate binding protein	2.5	3.0
CJA_3248	*agd31B*	α-glucosidase[g]	2.3	2.4
CJA_2610	*bgl2C*	β-galactosidase	2.2	2.6
CJA_1497	*cel3B*	β-glucosidase	2.2	2.3
CJA_0849	*cgs94A*	cyclic β-1,2-glucan synthetase	2.1	2.2
CJA_3263	*cgt13B*	cyclomaltodextrin transferase	2.1	2.4
CJA_0042	*pel1D*	pectate lyase	2.1	2.5
CJA_3281	*abf62A*	α-L-arabinofuranosidase	2.0	2.8
CJA_2769	*abf51A*	α-L-arabinofuranosidase	2.0	2.5
CJA_2770	*man26A*	*endo*-1,4-β mannanase	1.9	4.2
CJA_2616	*cbp2D*	carbohydrate binding protein	1.9	3.5
CJA_0044	*pel1C*	pectate lyase	1.9	2.7
CJA_0816	*gly43C*	β-xylosidase/α-L-arabinfuranosidase[e]	1.8	3.0
CJA_3066	*xyn10C*	*endo*-1,4-β-xylanase[h]	1.8	2.4
CJA_2040	*pel10B*	pectate lyase	1.7	2.7
CJA_1140	*cel3D*	cellodextrinase	1.7	3.0
CJA_0450	*axe2C*	acetyl xylan esterase[i]	1.7	3.8
CJA_0224	*glu16B*	β-glucanase	1.7	2.2
CJA_2707	*bgl35A*	β-galactosidase[f]	1.5	2.1
CJA_2872	*agd31A*	α-glucosidase	1.5	2.3
CJA_2887	*gla67A*	α-glucuronidase[j]	1.4	2.9
CJA_0350	*hex20A*	N-acetyl-β-hexosaminidase	1.4	2.5
CJA_0225	*glu16A*	β-glucanase	1.3	2.1
CJA_2993	*chi18C*	*endo*-chitinase	1.3	2.2
CJA_3279	*xyn5A*	*endo*-1,4-β-xylanase	1.3	2.3
CJA_3470	*man5C*	*endo*1,4-β-mannanase	1.3	2.1
CJA_3280	*xyn10B*	*endo*-1,4-β-xylanase[k]	1.3	3.2
CJA_3070	*gly43G*	β-xylosidase/α-L-arabinfuranosidase[e]	1.2	2.3
CJA_0559	*cbp35B*	carbohydrate binding protein	1.1	2.2

[a] RNASeq sampling performed in biological triplicate
[b] Gene names and predicted functions according to Deboy *et al.*[1]
[c] $\log_2$ scale
[d] $-\log_{10}$ conversion
[e] function confirmed by Cartmell, *et al.*
[f] function confirmed by Larsbrink, *et al.* [4]
[g] Function confirmed by Larsbrink, *et al.* [10]
[h] Function confirmed by Milward-Sadler, *et al.* [8]
[i] Function confirmed by Montainer, *et al.* [11]
[j] Function confirmed by Nagy, *et al*. [12]
[k] Function confirmed by Kellett, *et al.* [13]

Table S4. List of genes up-regulated during stationary phase compared to exponential phase on xyloglucan[a].

Locus ID	Gene[b]	Predicted Function[b]	Fold Change[c]	p-value[d]
CJA_1883	*gly57A*	glycoside hydrolase	4.5	2.5
CJA_1522	*amy13B*	α-amylase	2.0	3.2
CJA_1497	*cel3B*	β-glucosidase	1.7	2.4
CJA_1923	*acm73B*	*endo*-β-N-acetylglucosaminidase	1.7	2.3

[a] RNASeq sampling performed in biological triplicate
[b] Gene names and predicted functions according to Deboy *et al* [1]
[c] $\log_2$ scale
[d] $\log_{10}$ conversion

Table S5. Identity matrix between *Cj*GH5_4 enzymes and other specific previously characterized endo-xyloglucanases[a].

	***Cj*GH5D**[b]	***Cj*GH5E**[c]	***Cj*GH5F**[d]	***Bo*GH5**[e]	***Pp*XG5**[f]	**XEG5A**[g]
***Cj*GH5D**[b]	----	41%	41%	35%	35%	26%
***Cj*GH5E**[c]	41%	----	65%	41%	42%	28%
***Cj*GH5F**[d]	41%	65%	-----	45%	41%	29%
***Bo*GH5**[e]	35%	41%	45%	-----	33%	24%
***Pp*XG5**[f]	35%	42%	41%	33%	-----	29%
**XEG5A**[g]	26%	28%	29%	24%	29%	-----

[a]Only amino acid sequences of the catalytic domains were used in the analysis.
[b]CJA_3010 (ACE84905.1).
[c]CJA_3337 (ACE83841.1).
[d]CJA_2959 (ACE86198.1).
[e]*Bacteroides ovatus* GH5A (EDO11444.1).
[f]*Paenibacillus pabuli* XG5 (WP_017688986.1).
[g]XEG5A from rumen microflora metagenomics library (ACZ54907.1).

Table S6. Maximum growth and growth rate of *C. japonicus* strains using reduced or limiting concentrations of XyG[a].

	**Growth Rate[b]**	**Max. OD[c]**	**T1[d]**	**T2**
	**Glc 0.2%**			
**WT**	0.26 ± 0.01	0.81 ± 0.05	4	7
**Δ*xyl31A***	0.23 ± 0.01	0.81 ± 0.05	4	9
**Δ3010 Δ3337 Δ2959 Δ2477**	0.28 ± 0.03	0.83 ± 0.01	4	8
	**XyG 0.5%**			
**WT**	0.22 ± 0.04	1.11 ± 0.01	7	11
**Δ*xyl31A***	ND	0.19 ± 0.03	ND	ND
**Δ3010 Δ3337 Δ2959 Δ2477**	0.22 ± 0.01	1.18 ± 0.03	3	8
	**XyG 0.25%**			
**WT**	0.25 ± 0.06	1.32 ± 0.07	7	10
**Δ*xyl31A***	ND	0.19 ± 0.02	ND	ND
**Δ3010 Δ3337 Δ2959 Δ2477**	0.25 ± 0.03	1.22 ± 0.04	5	9
	**XyG 0.125%**			
**WT**	0.29 ± 0.03	0.47 ± 0.02	6	9
**Δ*xyl31A***	ND	0.11 ± 0.01	ND	ND
**Δ3010 Δ3337 Δ2959 Δ2477**	0.27 ± 0.02	0.48 ± 0.01	7	11

[a]Experiments were performed in biological triplicate; average and standard deviation reported.
[b]Growth rate is reported as generations per hour.
[c]Maximum growth as measured by optical density (OD) at 600 nm.
[d]Time points used to calculate growth rate: T1 (initial) and T2 (final).

Table S7. Primer sequences used for recombinant protein production.

Primer	Oligonucleotide Sequence[a]	Recombinant protein
*Cj*GH5D-Full-NheI-F	5´ - GACCGCTAGCATGTGTGGTAGCGCCGGTGGCGGCTC - 3´	*Cj*SRL-GH5D
*Cj*GH5D -XhoI-R	5´- GGTCCTCGAGTTATTGTGCTCCTGCGCCCTCC - 3´	
*Cj*GH5D-NheI- F	5´ -GACCGCTAGCGGGCTTTATCCCAGTTACAACACC - 3´	*Cj*GH5D
*Cj*GH5D -XhoI-R	5´- GGTCCTCGAGTTATTGTGCTCCTGCGCCCTCC - 3´	
*Cj*GH5E-Full-NheI-F	5´ - GACCGCTAGCATGCAAACAGCCAGTTGTAAGTATG - 3´	*Cj*CBM2-CBM10-
*Cj*GH5E -Full-XhoI-R	5´ - GGTCCTCGAGTCAGAAGGTTGCGTTTACAATCG - 3´	GH5E
*Cj*GH5E-LIC-F	5´ -TACTTCCAATCCAATGCCATGCTGACCAGTGTGGAGTTAACGCGC- 3´	*Cj*GH5E
*Cj*GH5E –LIC-R	5´ -TTATCCACTTCCAATGTTATCAGAAGGTTGCGTTTACAATCGC- 3´	
*Cj*GH5F-Full-NheI-F	5´ - GACCGCTAGCATGCAGAATTGCGGCAGCGGTGGCG - 3´	*Cj*FN3-GH5F
*Cj*GH5F -XhoI-R	5´ - GGTCCTCGAGTTATTGCGCCGCATTGATAATGG - 3´	
*Cj*GH5F-NheI- F	5´ - GACCGCTAGCAGTGTGCAATTGGCCAGGTTGATG - 3´	*Cj*GH5F
*Cj*GH5F -XhoI-R	5´ - GGTCCTCGAGTTATTGCGCCGCATTGATAATGG - 3´	

[a]Underlined sequences are the restriction sites.

Table S8. Primers used for generation of in-frame deletion mutants.

Primer name[a]	Sequence	Source
CJA_3010 UP (5')	GCTATGACATGATTACGAATTCCCAGCGCGATAAAGAGCAG	This study
CJA_3010 UP (3')	GTTCATCATTTACACCGCTCCATTGCC	This study
CJA_3010 DOWN (5')	CAGCGGTGTAAATGATGAACAACTCCATCGCCTATATC	This study
CJA_3010 DOWN (3')	CGACGGCCAGTGCCAAGCTTACCCACCGTCACTGTTAAAACG	This study
CJA_3010 INT (5')	GGTGGTGAATTCGCAGTTGGTCCAGC	This study
CJA_3010 INT (3')	GGTGGTTCTAGACAGGTCCATCTGCT	This study
CJA_3337 UP (5')	GCTATGACATGATTACGAATTCCGCGCTCTTGTGTTTGTAATCG	This study
CJA_3337 UP (3')	ATGGGGTGTCACATTAGCGTTATTCTCCGTTGACAC	This study
CJA_3337 DOWN (5')	CGCTAATGTGACAGGGCATTTGCCAGC	This study
CJA_3337 DOWN (3')	CAGTGCCAAGCTTTGGTGCAGGTGCTGTTA	This study
CJA_3337 INT (5')	GGTGGTGAATTCGGTGTCATCGTCCC	This study
CJA_3337 INT (3')	GGTGGTTCTAGAACGCCGGGTCAAT	This study
CJA_2959 UP (5')	GCTATGACATGATTACGAATTCAAACAAAATTACCCTGGTGC	This study
CJA_2959 UP (3')	CACGATATTACATTTTATTATTTTCCTTTAGCTGATGGATGGA	This study
CJA_2959 DOWN (5')	ATAATAAAATGTAATATCGTGCGGGAAAGCGTG	This study
CJA_2959 DOWN (3')	GCCAGTGCCAAGCTTAGAGAAATTAATTTCCAC	This study
CJA_2959 INT (5')	GGTGGTGAATTCCGACACCAGTGCG	This study
CJA_2959 INT (3')	GGTGGTTCTAGACACGGGGATGCGG	This study
CJA_2477 CONF (5')	AGGAAACCGGGTGTTAC	This study
CJA_2477 CONF (3')	GTACACCCTTGGGTA	This study
CJA_2477 INT (5')	TGAATGAATC CGTTGAGA	This study
CJA_2477 INT (3')	GTAAGGCACTAATAGCCG	This study

[a] *cel5D* (CJA_3010) encodes *Cj*GH5D, *cel5E* (CJA_3337) encodes *Cj*GH5E, *cel5F* (CJA_2959) encodes *Cj*GH5F, and *gly74A* (CJA_2477) encodes *Cj*GH74A).

Table S9. X-ray data collection and refinement statistics for *Cj*GH5D.

	Apo-structure	XXXG-NHCOCH$_2$	GXLG
Data collection			
Space group	$P2_12_12_1$	$P2_12_12_1$	$P2_12_12_1$
Cell dimensions			
a, *b*, *c* (Å)	55.0, 96.4, 159.0	55.5, 97.6, 157.2	55.3, 95.8, 157.2
α, β, γ (°)	90.0, 90.0, 90.0	90.0, 90.0, 90.0	90.0, 90.0, 90.0
Resolution (Å)	82.58-1.60 (1.63-1.60)	82.92-2.10 (2.16-2.10)	81.96-1.90 (1.95-1.90)
R_{sym} or R_{merge}	0.060 (1.717)	0.059 (0.547)	0.084 (1.465)
R_{pim}	0.024 (0.695)	0.028 (0.256)	0.031 (0.592)
$CC_{1/2}$	0.999 (0.699)	0.998 (0.933)	0.998 (0.813)
I / σI	11.8 (1.0)	12.8 (2.7)	8.5 (0.8)
Completeness (%)	100.0 (100.0)	100.0 (100.0)	100.0 (100.0)
Redundancy	7.8 (7.9)	6.2 (6.3)	7.7 (7.6)
Refinement			
No. reflections	106661	48113	63393
R_{work} / R_{free}	0.14/0.20	0.22/0.28	0.23/0.30
No. atoms			
Protein	5936	5792	5843
Ligand/ion	200	178	168
Water	587	186	98
B-factors (Å^2)			
Protein	34	50	56
Ligand/ion	54	47	58
Water	45	47	44
R.m.s deviations			
Bond lengths (Å)	0.018	0.017	0.018
Bond angles (°)	1.7	1.8	1.9
Ramachandran plot residues			
In most favorable regions (%)	96.4	96.7	95.9
In allowed regions (%)	3.6	3.1	3.8
PDB code	5OYC	5OYD	5OYE

Supplemental Figures

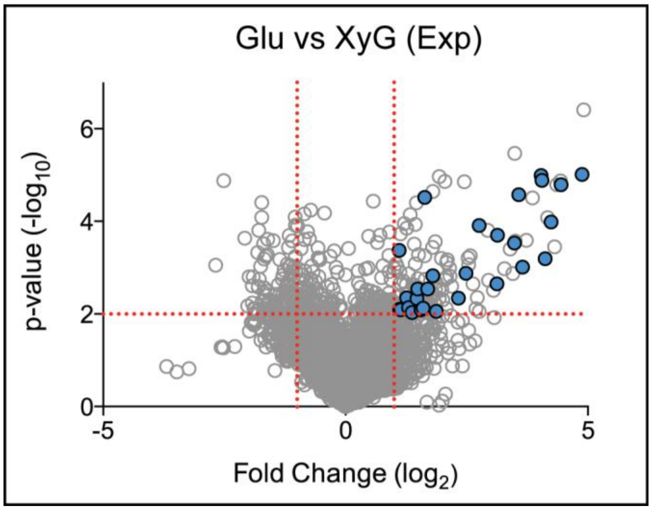

Figure S1. Volcano plots summarizing the RNAseq data for a comparative analysis of *C. japoncius* exponential phase cells grown on either glucose or xyloglucan. The volcano plots represents a comparison between exponentially growing cells (glucose vs xyloglucan). Each gray circle denotes a single gene, and the blue-filed circles indicate up-regulated CAZyme genes. The complete list of up-regulated CAZyme genes can be found in Table S1. Fold change in gene expression (log_2 scale) is plotted on the x-axis and p-value ($-log_{10}$ scale) is plotted on the y-axis. For orientation on the x-axis (fold change), positive values indicate genes that are up-regulated when grown using xyloglucan as the sole carbon source. The red dashed lines indicate significance cut-off values (2-fold for gene expression and p-value of 0.01).

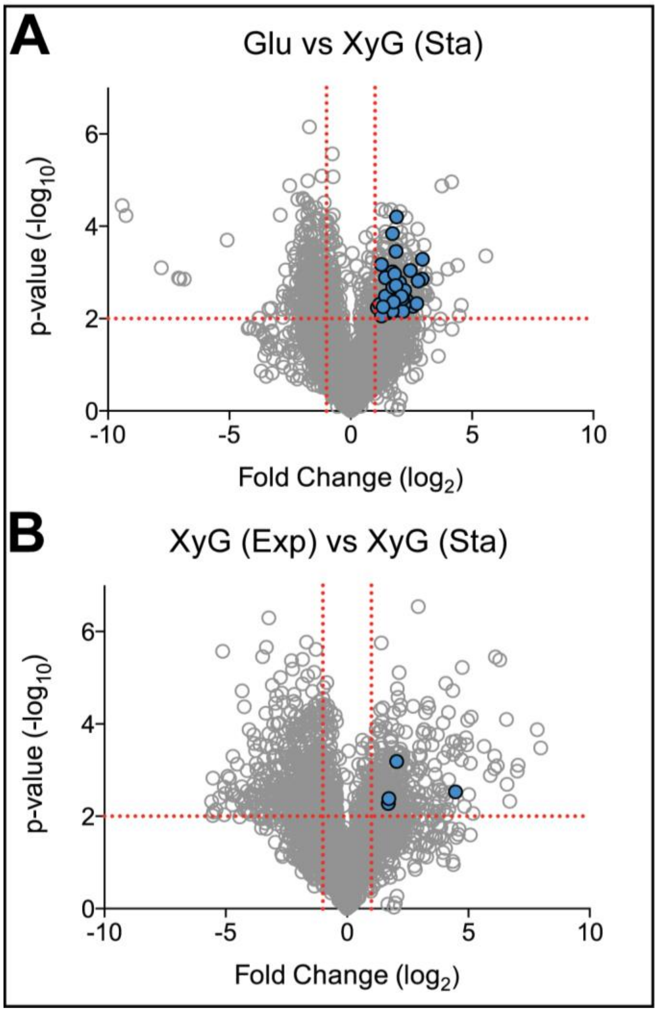

Figure S2. Volcano plots summarizing transcriptomic analysis of *C. japoncius* cells grown on either glucose or xyloglucan. The volcano plots represent comparisons between **A)** stationary phase cells (glucose vs xyloglucan), or **B)** xyloglucan grown cells in either exponential or stationary phase. Each gray circle denotes a single gene, and the blue-filed circles indicate up-regulated CAZyme genes. The complete list of up-regulated CAZyme genes for panel A can be found in Table S3, and for panel B in Table S4. The fold change (log_2 scale) is plotted on the x-axis and the p-value (-log_{10} scale) is plotted on the y-axis. For orientation on the x-axis, positive values indicate genes that are up-regulated when grown using xyloglucan as the sole carbon source for panel A, and genes up-regulated during stationary phase for panel B. The red dashed lines indicate the significance cut-off values (2-fold for gene expression and p-value of 0.01).

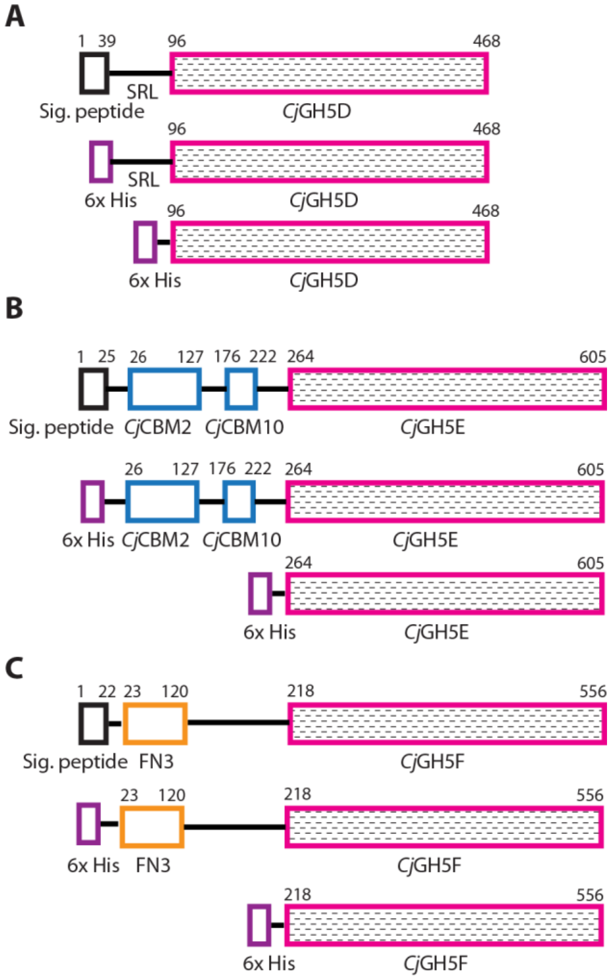

Figure S3. Modular architecture of the native *Cj*GH5_4 enzymes with the different expression constructs used in the current study. A) The locus CJA_3010 (GenBank ACE84905.1) encodes a signal peptide, a serine rich linker, and a GH5 catalytic domain (*Cj*GH5D). **B)** CJA_3337 (GenBank ACE83841.1) encodes a signal peptide, two carbohydrate binding modules (CBM2 and CBM10), and a GH5 catalytic domain (*Cj*GH5E). **C)** CJA_2959 (GenBank ACE86198.1) encodes a signal peptide, an FN3 domain, an X181 module, and a GH5 catalytic domain (*Cj*GH5F). All expression constructs were designed to produce 6x His-Tag at the N-terminus of the recombinant protein.

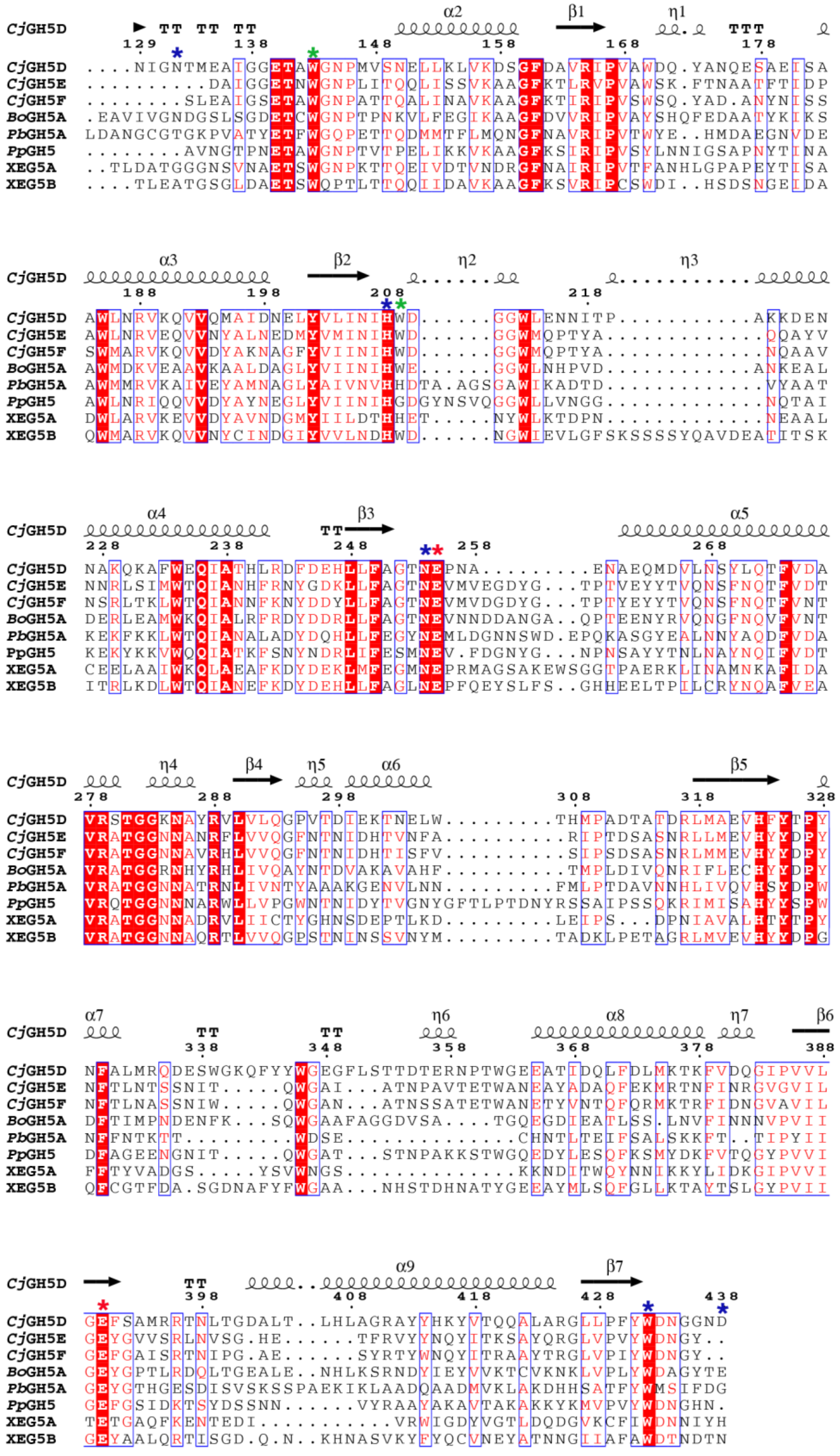
CjGH5D ....NIGNTMEAIGGETAWGNPMVSNELLKLVKDSGFDAVRIPVAWDQ.YANQESAEISA
CjGH5E ..........DAIGGETNWGNPLITQQLISSVKAAGFKTLRVPVAWSK.FTNAATFTIDP
CjGH5F ........SLEAIGSETAWGNPATTQALINAVKAAGFKTIRIPVSWSQ.YAD.ANYNISS
BoGH5A .EAVIVGNDGSLSGDETCWGNPTPNKVLFEGIKAAGFDVVRIPVAYSHQFEDAATYKIKS
PbGH5A LDANGCGTGKPVATYETFWGQPETTQDMMTFLMQNGFNAVRIPVTWYE..HMDAEGNVDE
PpGH5 ........AVNGTPNETAWGNPTVTPELIKKVKAAGFKSIRIPVSYLNNIGSAPNYTINA
XEG5A ..TLDATGGGNSVNAETSWGNPKTTQEIVDTVNDRGFNAIRIPVTFANHLGPAPEYTISA
XEG5B ....TLEATGSGLDAETSWQPTLTTQQIIDAVKAAGFKSVRIPCSWDI..HSDSNGEIDA
CjGH5D AWLNRVKQVVQMAIDNELYVLINIHWD......GGWLENNITP...........AKKDEN
CjGH5E AWLNRVEQVVNYALNEDMYVMINIHWD......GGWMQPTYA.............QQAYV
CjGH5F SWMARVKQVVDYAKNAGFYVIINIHWD......GGWMQPTYA.............NQAAV
BoGH5A AWMDKVEAAVKAALDAGLYVIINIHWE......GGWLNHPVD...........ANKEAL
PbGH5A AWMMRVKAIVEYAMNAGLYAIVNVHHDTA.AGSGAWIKADTD.............VYAAT
PpGH5 AWLNRIQQVVDYAYNEGLYVIINIHGDGYNSVQGGWLLVNGG.............NQTAI
XEG5A DWLARVKEVVDYAVNDGMYIILDTHHET.....NYWLKTDPN.............NEAAL
XEG5B QWMARVKQVVNYCINDGIYVVLNDHWD......NGWIEVLGFSKSSSSYQAVDEATITSK
CjGH5D NAKQKAFWEQIATHLRDFDEHLLFAGTNEPNA.........ENAEQMDVLNSYLQTFVDA
CjGH5E NNRLSIMWTQIANHFRNYGDKLLFAGTNEVMVEGDYG...TPTVEYYTVQNSFNQTFVDT
CjGH5F NSRLTKLWTQIANNFKNYDDYLLFAGTNEVMVDGDYG...TPTYEYYTVQNSFNQTFVNT
BoGH5A DERLEAMWKQIALRFRDYDDRLLFAGTNEVNNDDANGA..QPTEENYRVQNGFNQVFVNT
PbGH5A KEKFKKLWTQIANALADYDQHLLFEGYNEMLDGNNSWD.EPQKASGYEALNNYAQDFVDA
PpGH5 KEKYKKVWQQIATKFSNYNDRLIFESMNEV.FDGNYG...NPNSAYYTNLNAYNQIFVDT
XEG5A CEELAAIWKQLAEAFKDYDEKLMFEGMNEPRMAGSAKEWSGGTPAERKLINAMNKAFIDA
XEG5B ITRLKDLWTQIANEFKDYDEHLLFAGLNEPFQEYSLFS..GHHEELTPILCRYNQAFVEA
CjGH5D VRSTGGKNAYRVLVLQGPVTDIEKTNELW.........THMPADTATDRLMAEVHFYTPY
CjGH5E VRATGGNNANRFLVVQGFNTNIDHTVNFA..........RIPTDSASNRLLMEVHYYDPY
CjGH5F VRATGGNNAVRHLVVQGFNTNIDHTISFV.........SIPSDSASNRLMMEVHYYDPY
BoGH5A VRATGGRNHYRHLIVQAYNTDVAKAVAHF.........TMPLDIVQNRIFLECHYYDPY
PbGH5A VRATGGNNATRNLIVNTYAAAKGENVLNN.........FMLPTDAVNNHLIVQVHSYDPW
PpGH5 VRQTGGNNNARWLLVPGWNTNIDYTVGNYGFTLPTDNYRSSAIPSSQKRIMISAHYYSPW
XEG5A VRATGGNNADRVLIICTYGHNSDEPTLKD.........LEIPS...DPNIAVALHTYTPY
XEG5B VRATGGNNAQRTLVVQGPSTNINSSVNYM.........TADKLPETAGRLMVEVHYYDPG
CjGH5D NFALMRQDESWGKQFYYWGEGFLSTTDTERNPTWGEEATIDQLFDLMKTKFVDQGIPVVL
CjGH5E NFTLNTSSNIT.....QWGAI...ATNPAVTETWANEAYADAQFEKMRTNFINRGVGVIL
CjGH5F NFTLNASSNIW.....QWGAN...ATNSSATETWANETYVNTQFQRMKTRFIDNGVAVIL
BoGH5A DFTIMPNDENFK....SQWGAAFAGGDVSA....TGQEGDIEATLSS.LNVFINNNVPVII
PbGH5A NFFNTKTT.........WDSE.............CHNTLTEIFSALSKKFT..TIPYII
PpGH5 DFAGEENGNIT.....QWGAT...STNPAKKSTWGQEDYLESQFKSMYDKFVTQGYPVVI
XEG5A FFTYVADGS.....YSVWNGS.............KKNDITWQYNNIKKYLIDKGIPVVI
XEG5B QFCGTFDA.SGDNAFYFWGAA...NHSTDHNATYGEEAYMLSQFGLLKTAYTSLGYPVII
CjGH5D GEFSAMRRTNLTGDALT..LHLAGRAYYHKYVTQQALARGLLPFYWDNGGND
CjGH5E GEYGVVSRLNVSG.HE......TFRVYYNQYITKSAYQRGLVPVYWDNGY..
CjGH5F GEFGAISRTNIPG.AE......SYRTYWNQYITRAAYTRGLVPIYWDNGY..
BoGH5A GEYGPTLRDQLTGEALE..NHLKSRNDYIEYVVKTCVKNKLVPLYWDAGYTE
PbGH5A GEYGTHGESDISVSKSSPAEKIKLAADQAADMVKLAKDHHSATFYWMSIFDG
PpGH5 GEFGSIDKTSYDSSNN......VYRAAYAKAVTAKAKKYKMVPVYWDNGHN.
XEG5A TETGAQFKENTEDI...........VRWIGDYVGTLDQDGVKCFIWDNNIYH
XEG5B GEYAALQRTISGD.Q.N..KHNASVKYFYQCVNEYATNNGIIAFAWDTNDTN

Figure S4. **Amino acid sequence alignment showing regions of structural similarity between the catalytic domains of *Cellvibrio japonicus* GH5_4 enzymes (*Cj*GH5D, *Cj*GH5E, and *Cj*GH5F) and other members exhibiting endo-xyloglucanase activity belonging to the same family.**
Cellvibrio japonicus GH5s (*Cj*GH5D, Accession: ACE84905.1; *Cj*GH5E, Accession: ACE83841.1; and *Cj*GH5F, Accession: ACE86198.1); *Bacteroides ovatus* GH5 (*Bo*GH5A, Accession: WP_004298445.1); *Prevotella bryantii* B14 GH5 (*Pb*GH5A, Accession: EFI71705.1); *Paenibacillus Pabuli* GH5 (*Pp*GH5, PDB: 2JEP_A); ruminal metagenomic GH5s (XEG5A, Accession: ACZ54907.1; and XEG5B, Accession: ADB44000.1). Secondary structural elements are shown for *Cj*GH5D, with η referring to 310-helices, and α to α-helices (displayed as small and medium squiggles respectively), and β-strands shown as arrows, with TT and TTT representing strict β-turns and strict α-turns respectively. Alignment was created using Clustal Omega and Espript 3.0. Catalytic residues are marked with red asterisks, *Cj*GH5D active site residues interacting with the ligands (GXLG and XXXG-NHCOCH$_2$Br) via hydrogen bond formation and stacking interactions are marked with blue and green asterisks, respectively.

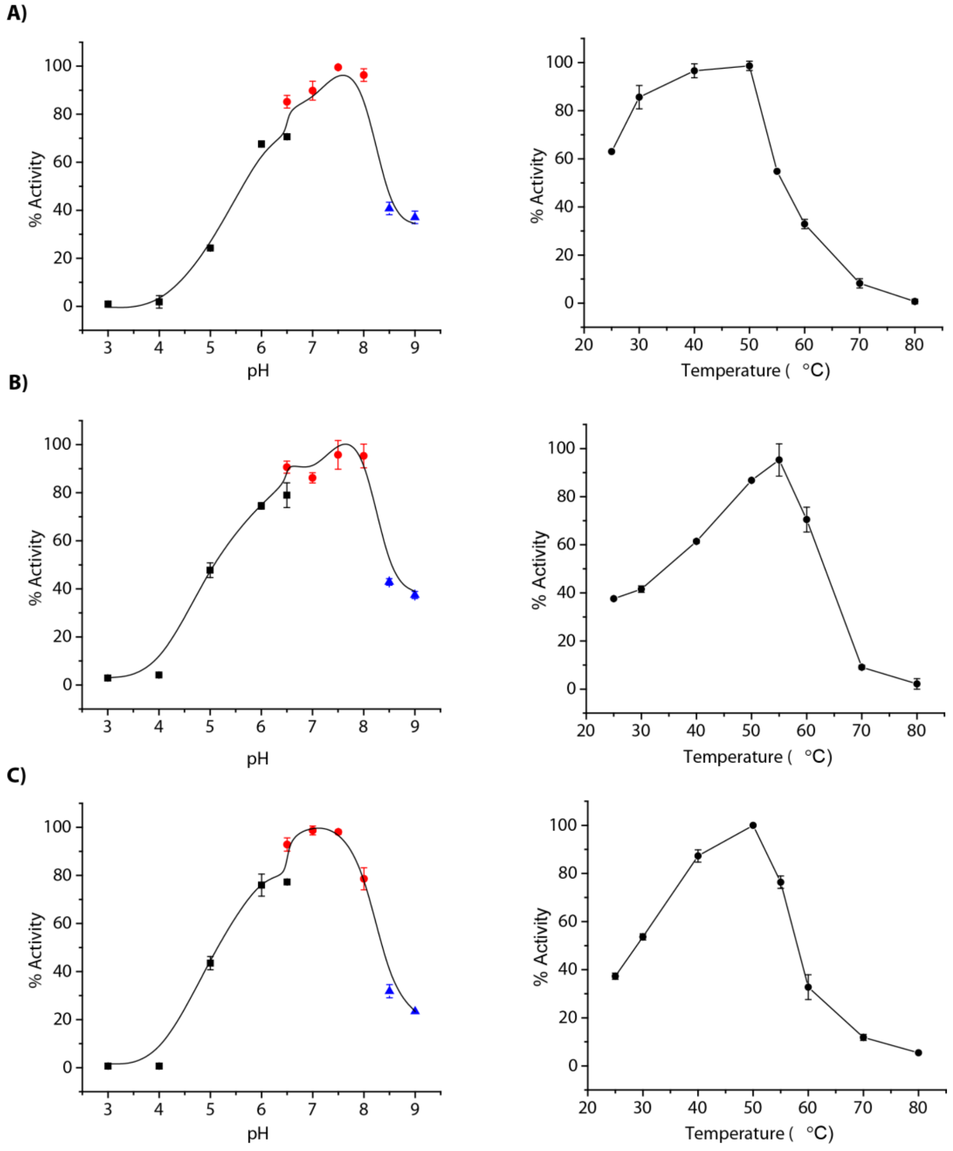

Figure S5. **pH and temperature profiles of *Cj*GH5_4 enzymes with tamarind seed XyG as a substrate**. **A)** *Cj*GH5D. **B)** *Cj*GH5E. **C)** *Cj*GH5F. Left panels are pH rate profiles while right panels are temperature profiles. Black squares, citrate buffer; red circles, phosphate buffer; and blue triangle, glycine buffer. Error bars represent standard error of the mean for 3 replicates. Lines were drawn to guide the eye with no physical significance.

A)

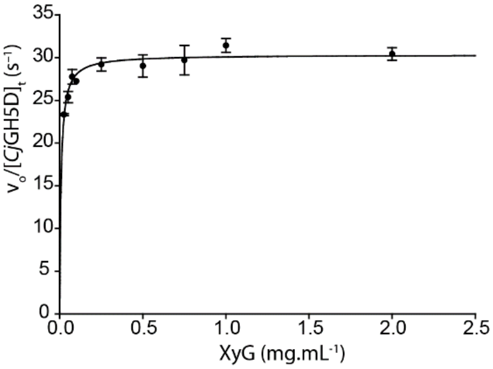

B)

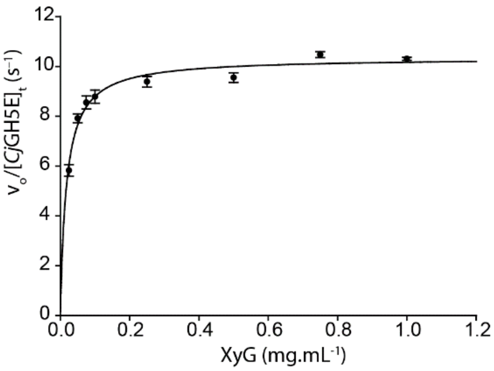

C)

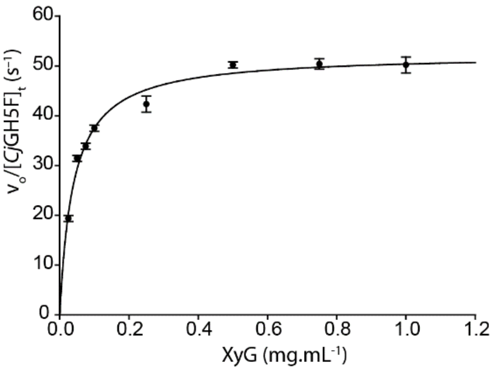

Figure S6. Michaelis-Menten kinetics of *Cj*GH5_4 enzymes on tamarind seed XyG. **A)** *Cj*GH5D. **B)** *Cj*GH5E. **C)** *Cj*GH5F. Error bars represent standard error based on 3 replicates.

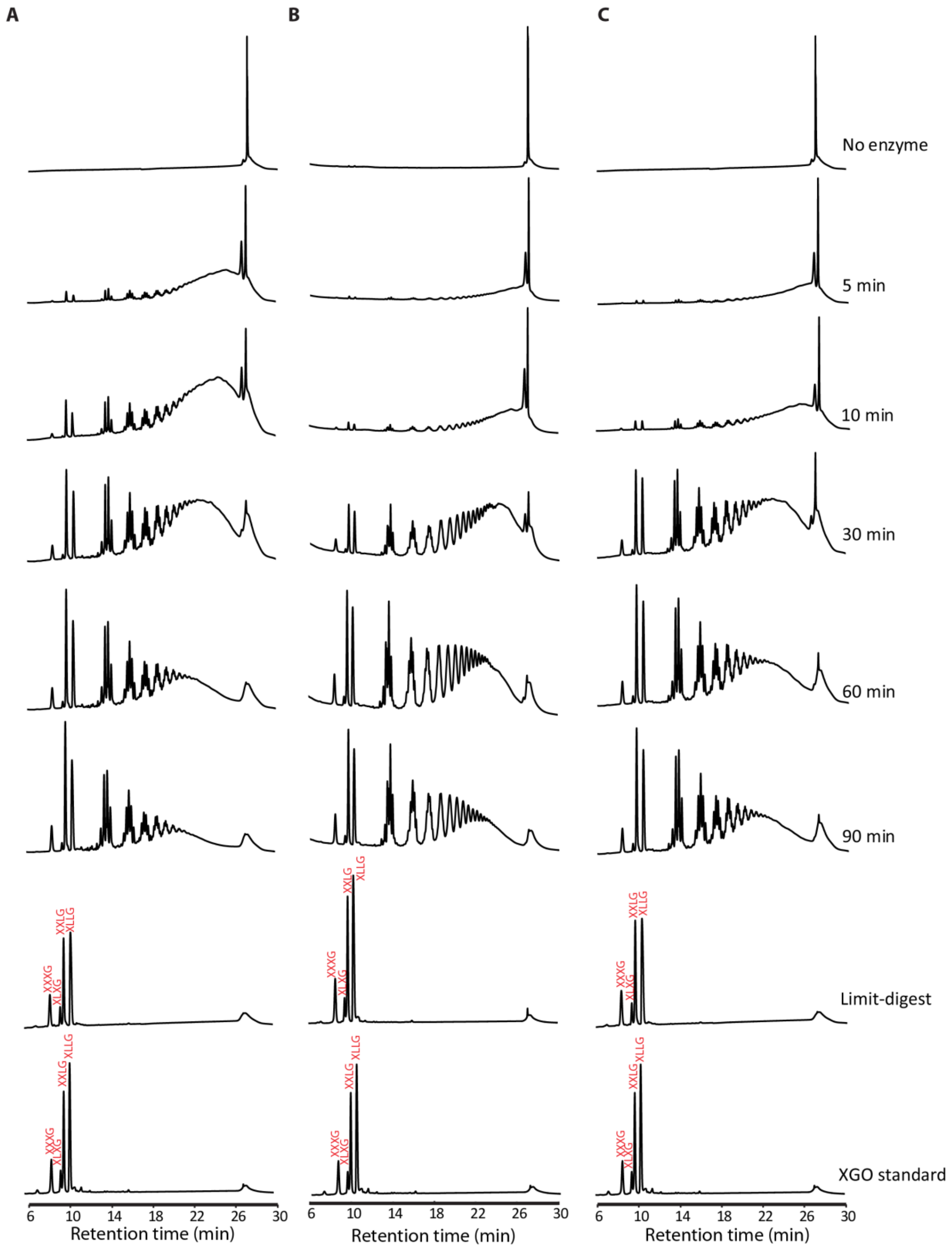

Figure S7. HPAEC-PAD analysis of the hydrolysis time course and limit-digest of *Cj*GH5_4 enzyme-xyloglucan degradation products. **A)** *Cj*GH5D. **B)** *Cj*GH5E. **C)** *Cj*GH5F.

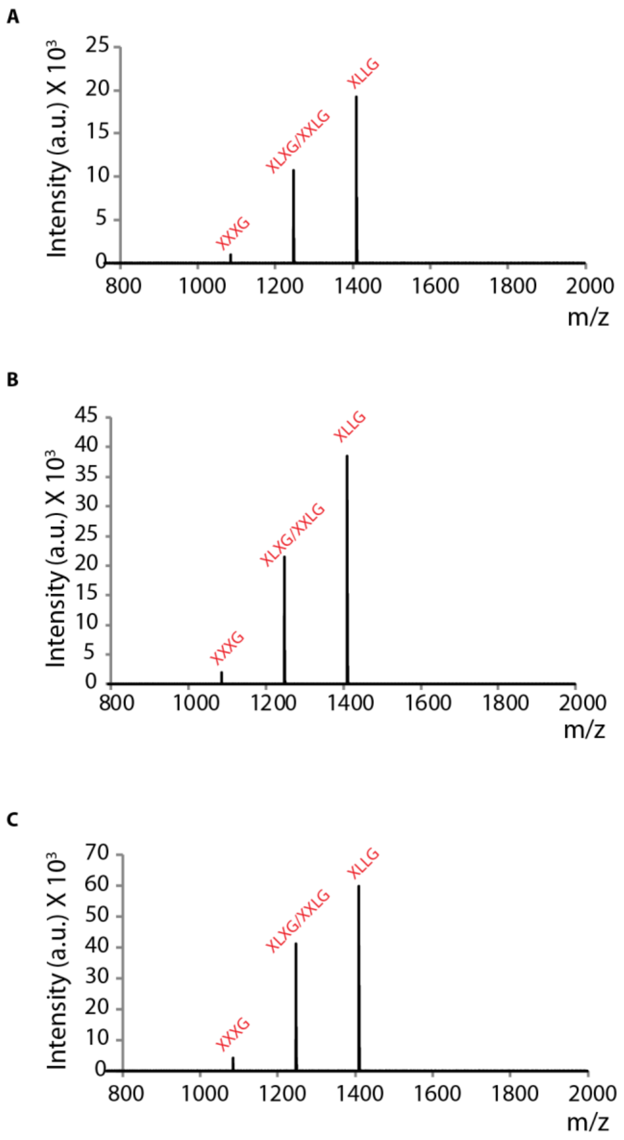

Figure S8. **MALDI-TOF analysis of the limit digest products of *Cj*GH5_4 enzymes upon incubation with tamarind seed XyG. A)** *Cj*GH5D. **B)** *Cj*GH5E. **C)** *Cj*GH5F. The observed molecular masses of the major 3 peaks were 1085.21, 1247.29 and 1409.37; which correspond to $[M+Na]^+$ of XXXG (calculated: 1085.9), XLXG/XXLG (calculated: 1248.05), and XLLG (calculated: 1410.19), respectively.

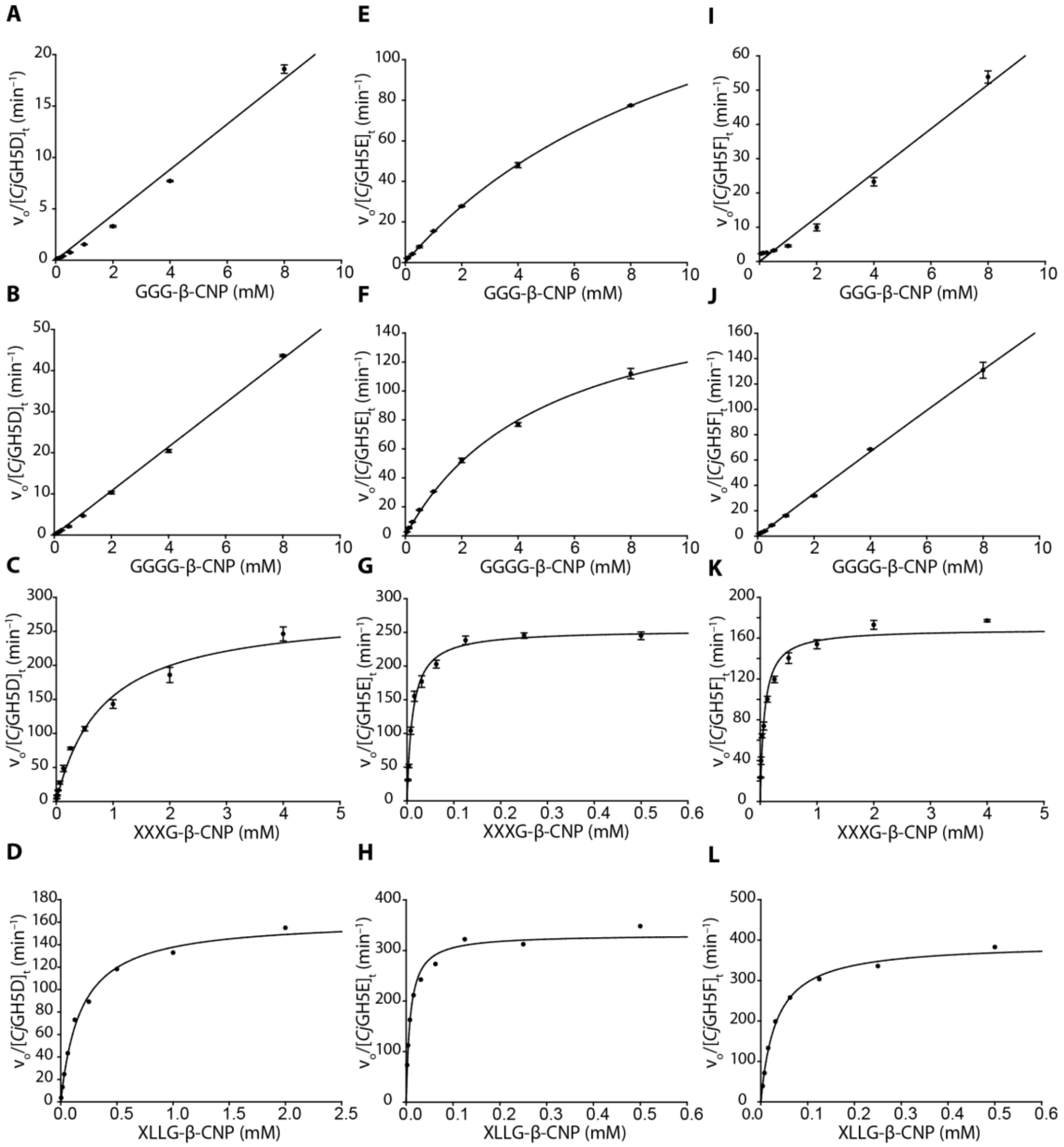

Figure S9. Michaelis-Menten kinetics of *Cj*GH5_4 enzymes on a panel of chromogenic (xylo)gluco-oligosaccharide glycosides. A-D) *Cj*GH5D. **E-H)** *Cj*GH5E. **I-L)** *Cj*GH5F. Error bars represent standard errors of the mean for 2 replicates. Only one replicate was done on XLLG-β-CNP due to limited availability of the substrate.

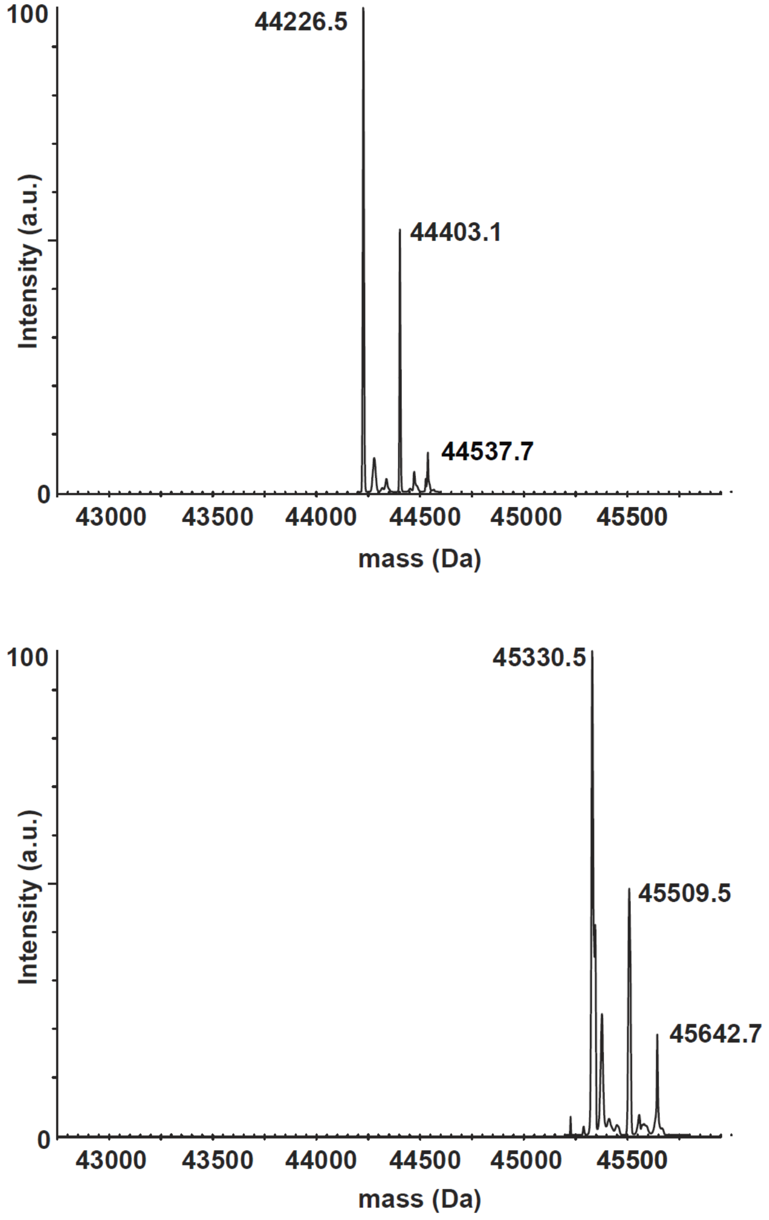

Figure S10. Intact protein mass spectrometry of CjGH5D with the XXXG-NHCOCH2Br inhibitor. A) *Cj*GH5D negative control with no inhibitor. **B)** *Cj*GH5D incubated with 2.5 mM of the inhibitor for 3 hours at 37 °C. The peak at 44226.5 Da corresponds to *Cj*GH5D (calculated 44222.2 Da) and 45330.5 Da to *Cj*GH5D-inhibitor covalent adduct (calculated: 45325.2 Da). Peak at 44403.1 Da (and correspondingly at 45509.5 Da) is attributed to the post translationally modified protein due to the N-gluconylation of the His-tag of the recombinant protein [14].

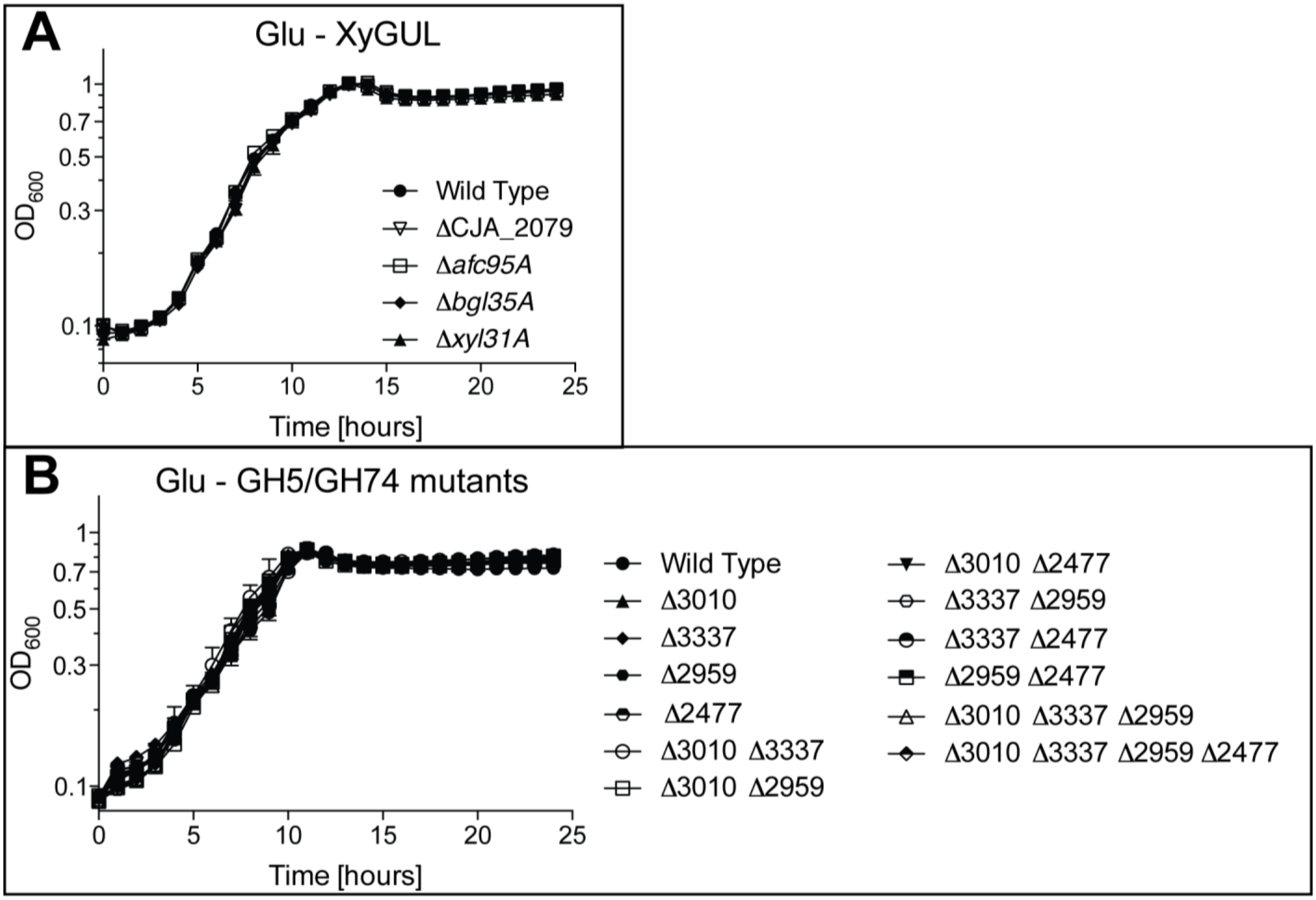

Figure S11. **Growth analysis control experiments for in-frame deletions mutants of the GH5_4, and GH74 genes on glucose.** Cultures were grown for 24 hours at 30°C with high aeration (200 RPM) in MOPS defined media supplemented with 0.5% (w:v) glucose as the sole carbon source. Graphs represent the average of three biological replicates and error bars represent the standard deviation, **A)** XyGUL mutants, **B)** single, double, triple, and quadruple deletion mutants of the GH5_4 and GH74 genes; CJA_3010 encodes *Cj*GH5D, CJA_3337 encodes *Cj*GH5E, CJA_2959 encodes *Cj*GH5F, and CJA_2477 encodes *Cj*GH74A.

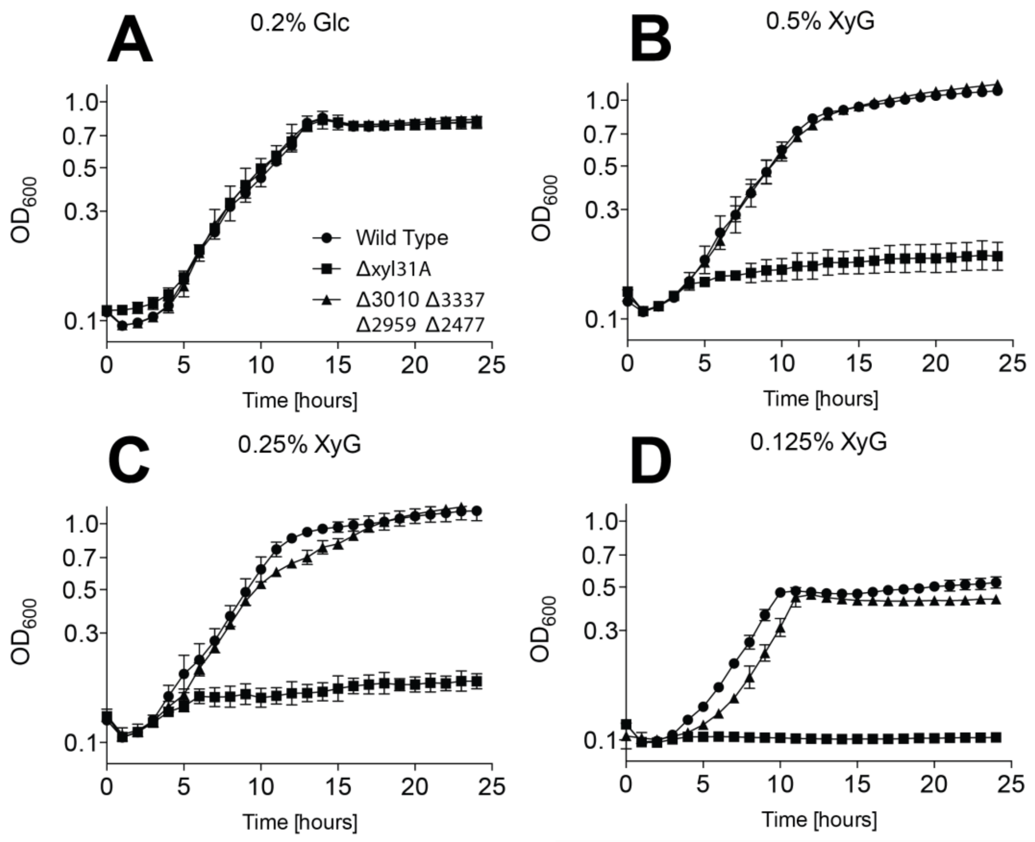

Figure S12. Growth analysis of *C. japonicus* strains using reduced or limiting concentrations of XyG. Wild type, a Δ*xyl31A* strain, and the quadruple mutant strain were grown using **A)** 0.2% glucose, **B)** 0.5% xyloglucan, **C)** 0.25% xyloglucan, or **D)** 0.125% xyloglucan as the sole carbon source. Standard deviation from biological triplicate experiments is shown. Maximum OD and growth rate calculations can be found in Table S6.

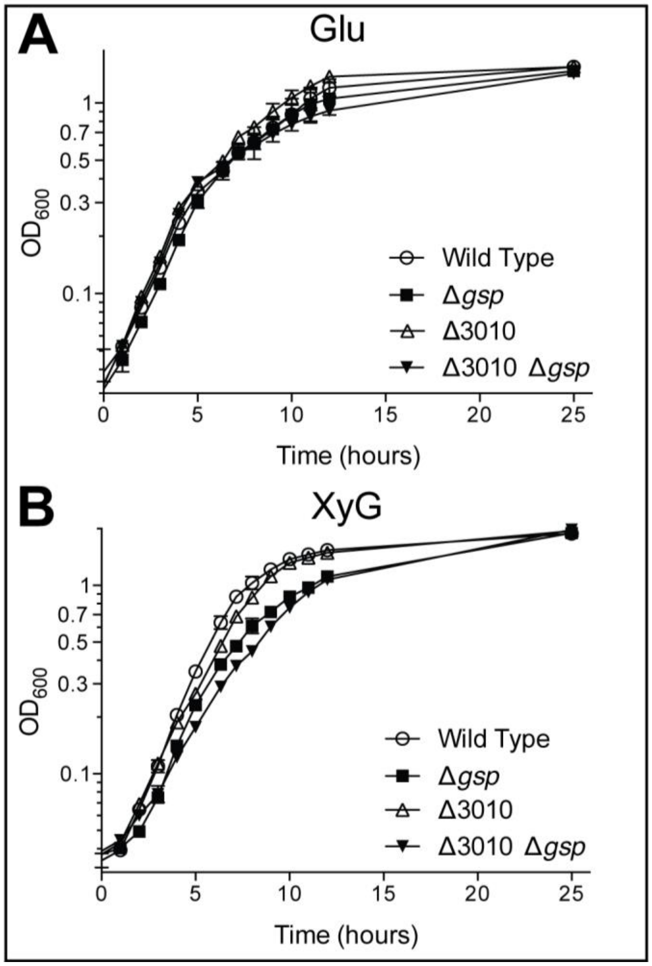

Figure S13. Growth analysis of ΔCJA_3010 and Δ*gsp* mutant strains when using glucose or xyloglucan. Cultures were grown in 18 mm test tubes at 30°C with shaking at 200 RPM using MOPS minimal media supplemented with **A)** 0.25% (w:v) glucose or **B)** 0.5% (w:v) xyloglucan as the sole carbon source. Open circles represent wild type, Δ*gsp* is represented by closed squares, ΔCJA_3010 (encoding *Cj*GH5D) is represented by open triangles, and ΔCJA_3010 Δ*gsp* is represented by inverted closed triangles. Graphs depict the average of biological triplicate experiments, and the error bars represent the standard deviation.

Supplemental References

1. DeBoy, R. T., Mongodin, E. F., Fouts, D. E., Tailford, L. E., Khouri, H., Emerson, J. B., Mohamoud, Y., Watkins, K., Henrissat, B., Gilbert, H. J. & Nelson, K. E. (2008) Insights into plant cell wall degradation from the genome sequence of the soil bacterium Cellvibrio japonicus, *J Bacteriol*. **190**, 5455-63.
2. Larsbrink, J., Izumi, A., Ibatullin, F. M., Nakhai, A., Gilbert, H. J., Davies, G. J. & Brumer, H. (2011) Structural and enzymatic characterization of a glycoside hydrolase family 31 alpha-xylosidase from Cellvibrio japonicus involved in xyloglucan saccharification, *The Biochemical journal*. **436**, 567-80.
3. Cartmell, A., McKee, L. S., Pena, M. J., Larsbrink, J., Brumer, H., Kaneko, S., Ichinose, H., Lewis, R. J., Vikso-Nielsen, A., Gilbert, H. J. & Marles-Wright, J. (2011) The structure and function of an arabinan-specific alpha-1,2-arabinofuranosidase identified from screening the activities of bacterial GH43 glycoside hydrolases, *The Journal of biological chemistry*. **286**, 15483-95.
4. Larsbrink, J., Thompson, A. J., Lundqvist, M., Gardner, J. G., Davies, G. J. & Brumer, H. (2014) A complex gene locus enables xyloglucan utilization in the model saprophyte Cellvibrio japonicus, *Mol Microbiol*. **94**, 418-33.
5. Beylot, M., Emami, K., McKie, V. A. & Gilbert, H. J. (2001) *Pseudomonas cellulosa* expresses a single hydrolase family 51 arabinofuranosidase, *Biochemical Journal*. **358**, 599-605.
6. Braithwaite, K. L., Barna, T., Spurway, T. D., Charnock, S. J., Black, G. W., Hughes, N., Lakey, J. H., Virden, R., Hazlewood, G. P., Henrissat, B. & Gilbert, H. J. (1997) Evidence that galactanase A from Pseudomonas fluorescens subspecies cellulosa is a retaining family 53 glycosyl hydrolase in which E161 and E270 are the catalytic residues, *Biochemistry*. **36**, 15489-500.
7. Halstead, J. R., Fransen, M. P., Eberhart, R. Y., Park, A. J., Gilbert, H. J. & Hazlewood, G. P. (2000) alpha-Galactosidase A from Pseudomonas fluorescens subsp. cellulosa: cloning, high level expression and its role in galactomannan hydrolysis, *FEMS microbiology letters*. **192**, 197-203.
8. Millward-Sadler, S. J., Davidson, K., Hazlewood, G. P., Black, G. W., Gilbert, H. J. & Clarke, J. H. (1995) Novel cellulose-binding domains, NodB homologues and conserved modular architecture in xylanases from the aerobic soil bacteria Pseudomonas fluorescens subsp. cellulosa and Cellvibrio mixtus, *The Biochemical journal*. **312 (Pt 1)**, 39-48.
9. Gardner, J. G., Crouch, L., Labourel, A., Forsberg, Z., Bukhman, Y. V., Vaaje-Kolstad, G., Gilbert, H. J. & Keating, D. H. (2014) Systems biology defines the biological significance of redox-active proteins during cellulose degradation in an aerobic bacterium, *Molecular Microbiology*. **94**, 1121-1133.
10. Larsbrink, J., Izumi, A., Hemsworth, G. R., Davies, G. J. & Brumer, H. (2012) Structural enzymology of Cellvibrio japonicus Agd31B protein reveals alpha-transglucosylase activity in glycoside hydrolase family 31, *The Journal of biological chemistry*. **287**, 43288-99.
11. Montanier, C., Money, V. A., Pires, V. M., Flint, J. E., Pinheiro, B. A., Goyal, A., Prates, J. A., Izumi, A., Stalbrand, H., Morland, C., Cartmell, A., Kolenova, K., Topakas, E., Dodson, E. J., Bolam, D. N., Davies, G. J., Fontes, C. M. & Gilbert, H. J. (2009) The active site of a carbohydrate esterase displays divergent catalytic and noncatalytic binding functions, *PLoS biology*. **7**, e71.
12. Nagy, T., Nurizzo, D., Davies, G. J., Biely, P., Lakey, J. H., Bolam, D. N. & Gilbert, H. J. (2003) The alpha-glucuronidase, GlcA67A, of Cellvibrio japonicus utilizes the carboxylate and methyl groups of aldobiouronic acid as important substrate recognition determinants, *The Journal of biological chemistry*. **278**, 20286-92.
13. Kellett, L. E., Poole, D. M., Ferreira, L. M., Durrant, A. J., Hazlewood, G. P. & Gilbert, H. J. (1990) Xylanase B and an arabinofuranosidase from Pseudomonas fluorescens subsp. cellulosa contain identical cellulose-binding domains and are encoded by adjacent genes, *The Biochemical journal*. **272**, 369-76.
14. Geoghegan, K. F., Dixon, H. B. F., Rosner, P. J., Hoth, L. R., Lanzetti, A. J., Borzilleri, K. A., Marr, E. S., Pezzullo, L. H., Martin, L. B., LeMotte, P. K., McColl, A. S., Kamath, A. V. & Stroh, J. G. (1999) Spontaneous alpha-N-6-phosphogluconoylation of a "His tag" in Escherichia coli: The cause of extra mass of 258 or 178 Da in fusion proteins, *Analytical Biochemistry*. **267**, 169-184.
